# Ruthenium(II) Polypyridyl Complexes and Their Use as Probes and Photoreactive Agents for G-quadruplexes Labelling

**DOI:** 10.3390/molecules27051541

**Published:** 2022-02-24

**Authors:** Julie Jiang, Titouan Teunens, Jérôme Tisaun, Laura Denuit, Cécile Moucheron

**Affiliations:** 1 Laboratoire de Chimie Organique et Photochimie, Service de Chimie et PhysicoChimie Organiques, Université Libre de Bruxelles, Avenue F. D. Roosevelt 50-CP 160/08, 1050 Brussels, Belgium; julie.jiang@ulb.be (J.J.); titouan.teunens@ulb.be (T.T.); jerome.tisaun@ulb.be (J.T.); laura.denuit@ulb.be (L.D.); 2 Laboratoire de Chimie des Matériaux Nouveaux, Université de Mons, Place du Parc 20, 7000 Mons, Belgium

**Keywords:** ruthenium(II) complexes, G-quadruplexes, photoprobes, photoreactive agents, labelling

## Abstract

Due to their optical and electrochemical properties, ruthenium(II) polypyridyl complexes have been used in a wide array of applications. Since the discovery of the light-switch ON effect of [Ru(bpy)_2_dppz]^2+^ when interacting with DNA, the design of new Ru(II) complexes as light-up probes for specific regions of DNA has been intensively explored. Amongst them, G-quadruplexes (G4s) are of particular interest. These structures formed by guanine-rich parts of DNA and RNA may be associated with a wide range of biological events. However, locating them and understanding their implications in biological pathways has proven challenging. Elegant approaches to tackle this challenge relies on the use of photoprobes capable of marking, reversibly or irreversibly, these G4s. Indeed, Ru(II) complexes containing ancillary π-deficient TAP ligands can create a covalently linked adduct with G4s after a photoinduced electron transfer from a guanine residue to the excited complex. Through careful design of the ligands, high selectivity of interaction with G4 structures can be achieved. This allows the creation of specific Ru(II) light-up probes and photoreactive agents for G4 labelling, which is at the core of this review composed of an introduction dedicated to a brief description of G-quadruplex structures and two main sections. The first one will provide a general picture of ligands and metal complexes interacting with G4s. The second one will focus on an exhaustive and comprehensive overview of the interactions and (photo)reactions of Ru(II) complexes with G4s.

## 1. Introduction

**Figure 1:**
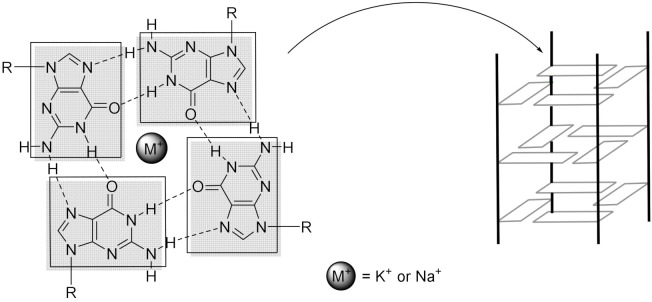
Structure of a G-tetrad and simplified schematic representation of a G-quadruplex.

When one thinks of DNA, one often thinks of the classical double-helix structure, B-DNA, that was discovered from X-ray data by Watson and Crick in 1953 [1]. In this form, two complementary and antiparallel strands are held together by hydrogen bonding between adenines and thymines or guanines and cytosines. Nevertheless, a wide variety of other structures exist such as the more commonly known A-DNA and Z-DNA or the less common ones such as i-motif [2,3] and four-way junctions [4,5] for example. Among them, G-quadruplexes (G4) are formed from guanine-rich sequences of DNA or RNA. In G-quadruplex structures, four guanines are associated by Hoogsten hydrogen bonds in a plane, which form a so-called G-quartet or tetrad, and a minimum of two tetrads stacked on top of each other is needed to form a G-quadruplex (Figure 1). These G4 structures can be stabilized by monovalent cations such as K^+^ or Na^+^ which can coordinate the oxygen of the carbonyl groups of guanines, as demonstrated in the 1960s by X-ray diffraction [6,7,8] and spectroscopic studies [9]. G-quadruplexes play an important role in gene expression [10,11] and their presence in the cell environment has been demonstrated at several sites and intensively studied at the telomeric ends of eukaryotic chromosomes [12,13]. At these telomeric ends, DNA is organized as a guanine-rich single strand rather than a double helix, which allows a favored quadruplex conformation and a privileged interaction with small molecules [14,15,16]. Bioinformatics studies have predicted the occurrence of quadruplex structures at many locations in the genome, particularly in promoter regions, or in genes involved in cell replication [17]. Computational studies have indicated that quadruplex structures may number as many as 700,000 [17,18,19].

Intensive research has been carried out over the past few decades to study G-quadruplexes as they have high potential to be used notably as therapeutic targets for cancer treatments thanks to their presence at the end of chromosomes [20]. They are crucial in cells as they play the role, among others, of cellular clock by shortening with each cell division, which finally brings cells to a state of senescence.

The stabilization of such G4 structures by small molecules or metal complexes is of key importance as this can inhibit the telomerase activity (activated in approximately 85–90% of cancer tumors), and thus, kill the cell through the induction of apoptosis. However, studying G-quadruplexes is not an easy task because they can adopt many different structures depending on the number and orientation of the strands that form the quadruplex, the stabilizing ions, or the sequence of nucleotides involved. Therefore, those structures will be briefly discussed in the next section. Afterwards a brief summary of the existing organic and metal complexes-based G-quadruplex ligands will showcase the great diversity of molecules able to interact with such G4. Lastly, an exhaustive presentation of ruthenium(II) polypyridyl complexes studied in presence of G-quadruplex structures will present their ability to interact and act as selective probes or potential anti-cancer agent which is the main focus of this review. 

### 1.1. G-quadruplex Structures

G-quadruplexes are nucleic acid sequences containing a repeat of guanines which form quartets, linked together to the rest of the sequence forming loops [21,22,23]. The sequence allowing such a conformation can be summarized as follows:

(1) $$G_{3-5}X_{B1}G_{3-5}X_{B2}G_{3-5}X_{B3}G_{3-5}$$

where the length and the composition of the sequence (*X*) that form the loops (*B*) vary from one structure to the other. A theoretical prediction model has been developed based on various G-quadruplex stability measurements. This model states that a structure can adopt a G4 conformation if it has a repeating unit comprising at least three consecutive guanines followed by at least one other base (usually several) [18,19].

(2) $$G_{\geq 3}X_{1-7}G_{\geq 3}X_{1-7}G_{\geq 3}X_{1-7}G_{\geq 3}X_{1-7}$$

G-quadruplexes can be formed from one to four different nucleic acid strands. They are generally defined by a stack of at least two guanine tetrads.

The guanine quartets form the centre of the quadruplex by means of Hoogsteen hydrogen bonds [22], while the loops are located on the outside of the structure. The grooves formed by this structure are stabilized by phosphodiester bonds. In the case of a single-stranded structure, the sequence can be folded back on itself in different ways. The loops can be diagonal, lateral, or inverted, which leads to different G4 conformations.

**Figure 2:**
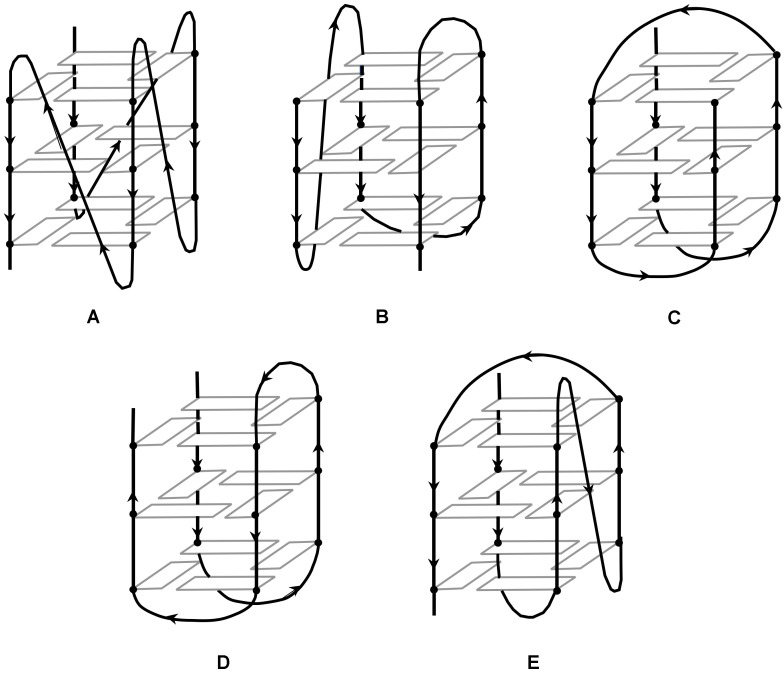
Schematic representation of various topologies adopted by simple unimolecular G-quadruplexes. (**A**)—Parallel conformation with three double chain reversal loops. (**B**)—Antiparallel conformation with one double chain reversal loop and two side loops. (**C**)—Antiparallel conformation with two side loops and a diagonal loop. (**D**)—Antiparallel conformation with three side loops. (**E**)—Antiparallel conformation with two diagonal loops and a double chain reversal loop [16,22].

The conformation adopted by a G4 structure will determine the direction of the strand base sequence. If a direction is arbitrarily defined, from top to bottom for example, it is possible to construct G4 structures in different ways (Figure 2). If all the guanine quartets participate in the top to bottom construction, a parallel structure will be spoken about (Figure 2A). If, on the other hand, half of the guanine quartets participate in the bottom-up construction, and the other half in the top-down construction, we speak of an antiparallel structure (Figure 2B–E). For single- or two-stranded G-quadruplexes, it is possible to observe a parallel conformation, implying that the top of the structure must be connected by a loop to the bottom of the structure for the adjacent strand. This is known as a propeller loop (Figure 2A) [24,25]. Another possibility is the antiparallel conformation, in which at least one of the strands is oriented in the opposite direction to the others. Although the loop can also exist in this type of structure, this implies the existence of another type of loop, connecting the upper (or lower) ends of the structures. Such a connecting loop between two adjacent vertices of posts is called a lateral loop (Figure 2B–D) [26,27,28]. When, on the other hand, the loop connects opposite posts, it will be called a diagonal loop (Figure 2C,E). Note that in this case, the direction of adjacent strands will alternate between parallel and antiparallel for steric reasons [29,30,31,32,33]. Furthermore, NMR studies have shown that guanines that do not constitute the quartet but are present in close proximity, for example in a loop, can participate in the stabilization of the overall structure [34].

The environment where the DNA strands are evolving has a major influence on the adopted conformation. The physiological environment controls the thermodynamic stability of the G-quadruplex due to the π-stacking existing by the induced conformation. The presence of positively charged ions that compensate for the overall negative charge of the DNA strand also plays a key role in the adopted structure. In particular, the presence of alkali ions such as Na^+^ or K^+^ will alter the parallel or antiparallel conformation of the quadruplex [35,36,37,38,39,40,41]. An NMR study published in 1993 showed that under sodium (Na^+^) conditions, the quadruplex adopts an antiparallel conformation with one diagonal and two lateral loops (Figure 2C) [42]. However, in potassium (K^+^) conditions, the sequence adopts a parallel conformation, with helical loops (propeller) (Figure 2A) [24,25]. The fact that the telomeric sequence can adopt a parallel conformation in potassium conditions was a surprise and led to numerous studies of quadruplexes in solution [35,36,37,38,39,40,43,44,45]. Because of the sensitivity of such systems to the environment, the conformations adopted by G-quadruplexes often coexist and easily switch from one to the other, suggesting that the two conformations are equi-energetic [35,39,46].

### 1.2. G-quadruplexes in Human Genome

#### 1.2.1. Telomeres and Their Functions

Telomeres are found at the ends of chromosomes and consist mainly of a single strand of DNA. Their presence protects the rest of the chromosome and allows the conservation of the genetic code. With each cell division, this single strand is shortened until it reaches a critical length that triggers the cell death mechanism. This regulation based on the progressive shortening of the telomere prevents older cells from accumulating too many mutations that could alter gene expression [47]. Telomerase is expressed or over-expressed in 80–85% of cancer cells and is not expressed in healthy somatic cells [48]. This enzyme acts as a regulator of telomere length, preventing them from shortening.

#### 1.2.2. Cellular Replication

The repeated sequence in human telomeres is a set of six bases: TTAGGG [49]. This sequence is accompanied by various proteins which have a necessary role for the protection of the telomere from the environment or in cell replication [50,51,52]. The function of the telomere is to protect the genetic information contained in the entire chromosome. Indeed, between 150 and 250 nucleotides are present at the 3′ end of the telomere sequence as a single strand and protect the rest of the chromosome from degradation or unwanted recombination [12]. Chromosomes are also protected by the presence and action of various proteins, among them hPOT1, a protein that interacts with TPP1, and whose role is to regulate the action of telomerase [53,54,55]. The absence of such proteins initiates the cell death response. The formation of more complex conformations such as G-quadruplexes competes with the action of these proteins, and stabilization of G4 structures by small molecules may also interfere with their action [56,57,58,59,60,61]. Studies have eventually determined that G-quadruplexes could present an anti-proliferative effect [62].

In a non-tumoral cell with a 24 h replication cycle, without an imported stabilizing molecule, a telomere of 5000 bases in length losing 100 bases per cycle would allow the cell to live between 40 and 50 days. In contrast, the use of molecules stabilizing G-quadruplex conformations shortens the lifetime of the cell drastically: only 7 to 10 days of life expectancy [63,64]. 

While the existence of G-quadruplex structures is no longer in question, their role in genetic processes is not well understood yet. Indeed, their presence in many gene promoter regions suggested that they might be of key importance in cell development, replication, and gene expression. In 2002, a study demonstrated that the transcription of *c-myc* (an oncogene) was adversely affected by the G4 conformation of the NHE III1 sequence upstream of the promoter sequence. In fact, if the sequence can retain its G-quadruplex conformation (by adding a stabilizer such as TMPyP4), *c-myc* is less expressed, whereas if the G4 conformation is dropped (by mutation for example), *c-myc* expression is favoured [65]. This first example was followed by many others [66,67]. 

## 2. Classification and Characteristics of G4 Ligands 

G-quadruplexes are dynamic structures in constant movement. In spite of this fact, there are lots of compounds capable of interacting with those ever-changing structures, and some of them can stabilize one or another conformation, leading to potential biological consequences.

**Figure 3:**
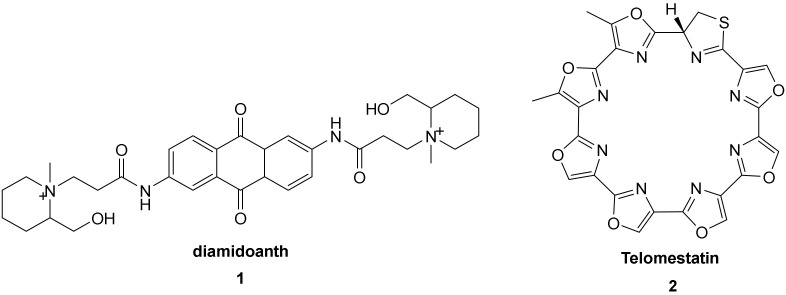
Structure of 2,6-bis[3-(4-methylpiperazino)proprionamido]-anthracene-9,10-dione **1** and telomestatin **2**.

Stabilization of G-quadruplex structures by small molecules was first demonstrated using an anthraquinone derivative **1** (Figure 3, diamidoanth) [68] and other studies have followed [20], mainly reporting the use of planar aromatic compounds, one end of which being positively charged [14,15,68]. Several studies have shown that the planar part of the molecule comes to rest on the external quartet, while intercalation between two guanine quartets has not been less observed so far [69,70,71,72,73,74]. Beyond the planar and positively charged ligands, telomestatin **2** (Figure 3) was of great interest to scientific research [75] and was the source of many more easily synthesized derivatives [76,77,78,79]. It has been shown that the size of the loops is important for molecular recognition [73]. 

**Figure 4:**
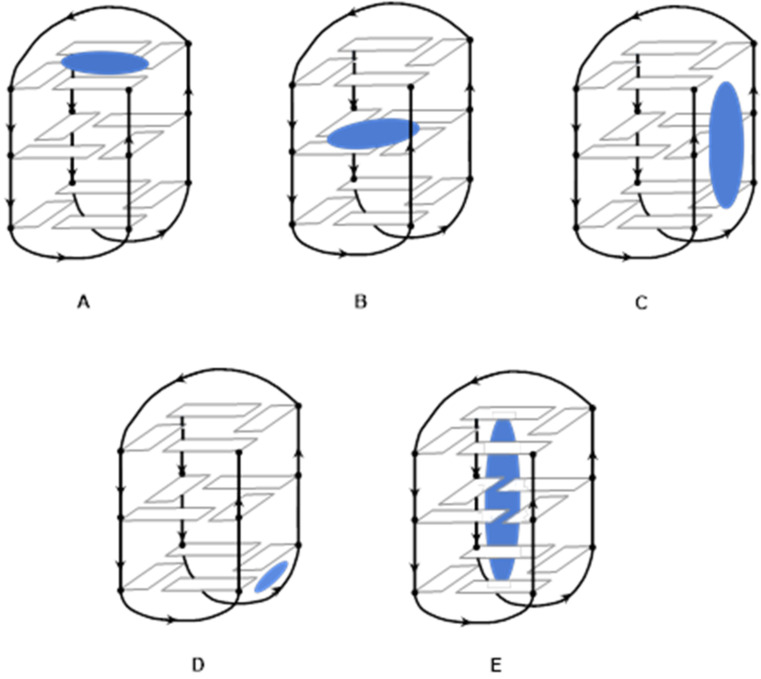
Interaction geometries between a G4-ligand and a G-quadruplex structure. (**A**)—(π-stacking) on an external quartet. (**B**)—intercalation between tetrads. (**C**)—interaction within a groove. (**D**)—interaction within a loop. (**E**)—interaction within the central channel.

G-quadruplexes present different interaction sites, among whom terminal quartets, internal quartets, grooves, loops, and the central channel (Figure 4) [16].

Ligands displaying aromatic rings mainly interact with the G-quadruplex by π-stacking on the terminal quartets. In comparison, ligands displaying some lateral chains preferably interact with the grooves and loops. The presence of a protonated amino group will help the ligand to interact with the negatively charged phosphate groups. Ligands displaying a long charged lateral chain will be able to interact through the central channel. All the ligands display at least one of those properties (abilities), but some ligands can interact with G4-structures by more than one geometry.

### 2.1. Organic G4 Ligands

#### 2.1.1. Stacking on the Terminal Tetrads

This interaction geometry is achieved by electrostatic, hydrophobic, and van der Waals interactions. The large aromatic surface resulting from the combination of four guanines allows better interaction with ligands displaying a large aromatic surface, making them more selective towards G4 structures than double-stranded DNA.

##### In Situ Protonated Ligands

While hydrophobicity must be sufficient to develop π-stacking with the external quartet, hydrophilia must also be present to allow the ligand to dissolve in water. This explains why several ligands have been designed to be protonated in experimental conditions.

**Figure 5:**
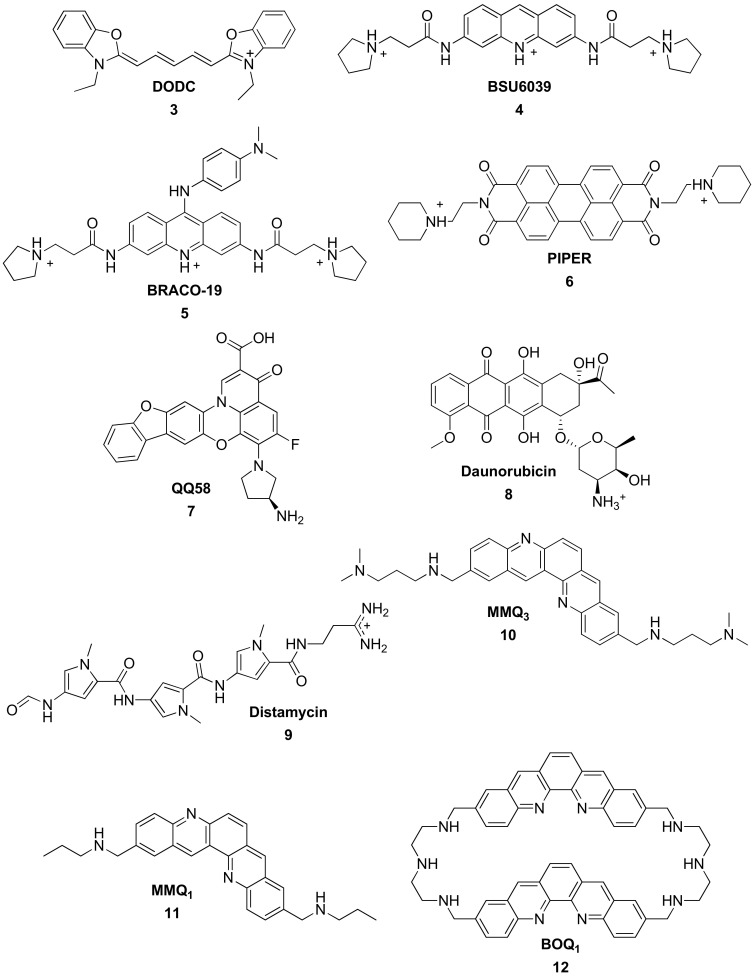

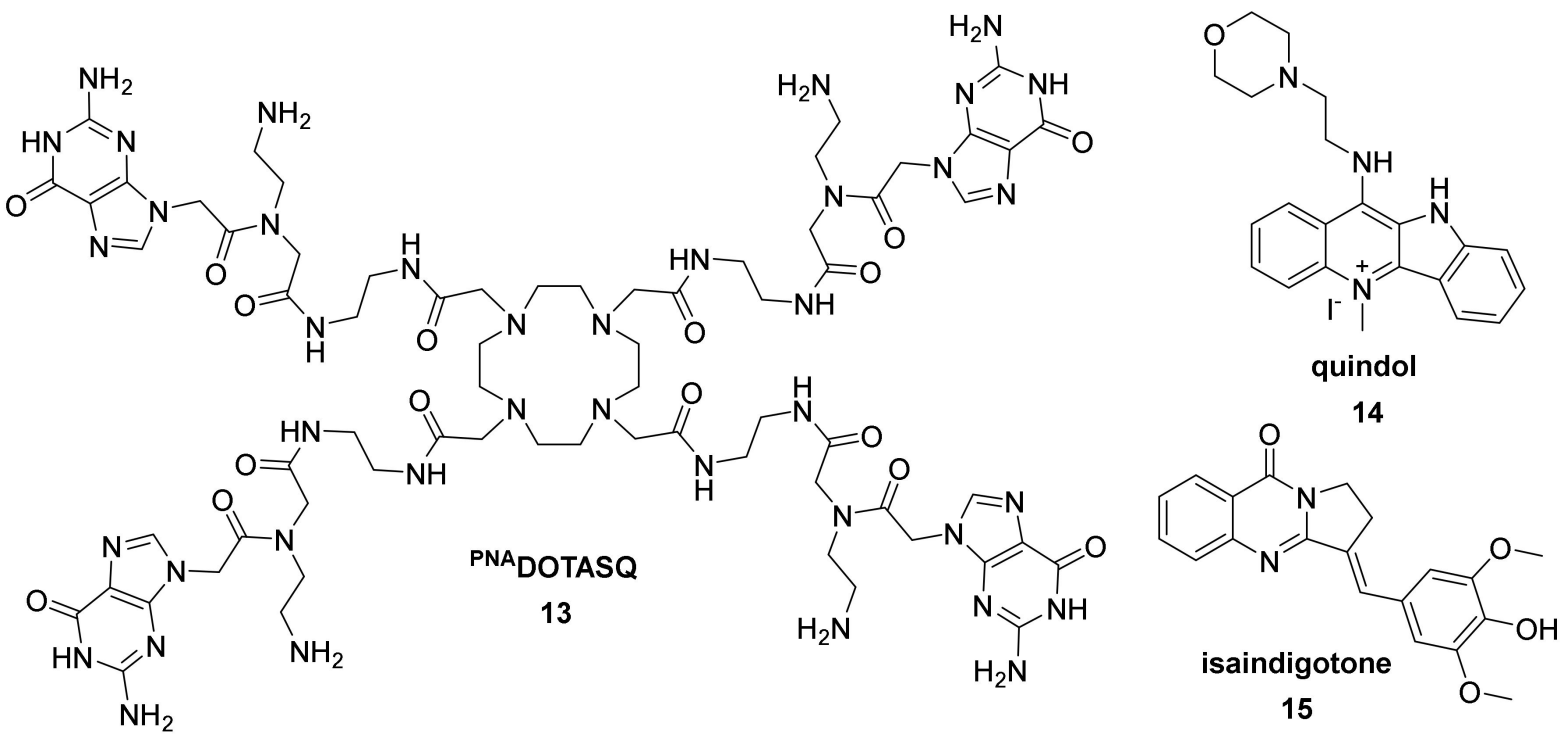
Structure of the in situ protonated ligands. **3**—DODC. **4**—BSU6039. **5**—BRACO-19. **6**—PIPER. **7**—QQ58. **8**—Daunorubicin. **9**—Distamycin. **10**—MMQ_3_. **11**—MMQ_1_. **12**—BOQ_1_. **13**—^PNA^DOTASQ in an open conformation. **14**—5-methyl-11-(2-morpholinoethylamino)-10H-indolo[3,2-b]quinolin-5-ium iodide (quindol). **15**—isaindigotone.

In 1996, Shafer showed that the relatively simple and small DODC (DODC = 3,3′-diethyloxadicarbocyanine) (Figure 5, compound **3**) interacts with G4 structures [80].

In 1997, Hurley developed a derivative of 2,6-diamidoanthraquinone (Figure 3, compound **1**) which exhibits an inhibitive behavior to telomerase [68].

However, it turned out that those compounds were not selective enough to G-quadruplexes, and focus moved to acridine derivatives [81,82]. BSU6039 (Figure 5, compound **4**) was proved to interact by π-stacking [70,83] and based on these results, BRACO-19 (Figure 5, **5**) was synthetized. This ligand is able to interact with three different grooves of G-quadruplexes [73,84,85,86,87] and showed encouraging results on the non-proliferation of cancerous cells [58,84,88,89,90].

Then, Hurley developed in 1998 a series of ligands with an even more extended planar aromatic moiety. In this context, perylene diimide (PIPER) (Figure 5, compound **6**) displays a more hydrophobic core but seems less aggressive to telomerase activity [69]. Later, some Iron(II) complexes bearing this PIPER ligand were also studied [91].

In the early 2000s, the family of quinobenzoxazines was studied. QQ58 (Figure 5, compound **7**) [92,93], a fluoroquinophenoxazine (FQP), showed an interesting cellular activity, namely the inhibition of polymerase stop assay, driven by the effective binding to G-quadruplexes via stacking on the external tetrads. Furthermore, this fluoroquinophenoxazine showed potent inhibition of telomerase activity with an IC_50_ value of 28 µM [92].

In the meantime, research also targeted compounds known for their affinity to duplex DNA. In this context, Daunorubicin [94,95] (Figure 5, compound **8**) was shown to strongly interact with G-quadruplexes. Three ligands can interact with only one G4 [96]. In comparison, two molecules of distamycin [97] (Figure 5, compound **9**) interact top to tail inside a groove or on the surface of the external tetrads [98,99,100,101,102]. This time the small aromatic surface is compensated by the presence of two ligands side by side.

Covering a large surface of the tetrad seems to be the top of the parameter list to ensure better interaction with the G-quadruplexes. Therefore, elbow-shaped pentacyclic quinacridine ligands were designed to cover more efficiently the guanines of the external quartet. Studies on MMQ_3_ (Figure 5, compound **10**) and MMQ_1_ (Figure 5, compound **11**) showed strong inhibition of telomerase activity [103] due to the interaction geometry with G4s involving the covering of three guanines. It also demonstrated the importance of the lateral chains interacting with the grooves [104]. Platinum(II) complexes bearing similar ligands were designed later [105].

A dimer of quinacridine (BOQ_1_) (Figure 5, compound **12**) was developed. The extended aromatic moieties allow it to interact more selectively with G-quadruplexes than with double-stranded DNA [106,107]. Another study showed that this compound could interact with the loops by adopting a half-closed conformation [108].

The dynamic change of conformation allows ^PNA^DOTASQ (Figure 5, compound **13**) to rearrange itself when in interaction with the G4 structure, on the surface of the external quartet [109].

Lots of alkaloids were studied as potential G4-ligands. For example, two molecules of quindoline (Figure 5, quindol **14**) stack on the external tetrads of telomeric G-quadruplexes, resulting in a high affinity for the G4 [110]. Finally, besides the characteristic end-stacking binding mode, isaindigotone (Figure 5, compound **15**) interacts preferably with the grooves than the loops. Four molecules of this ligand were proved to interact simultaneously with antiparallel G4 structures [111].

##### N-methylated Ligands

**Figure 6:**
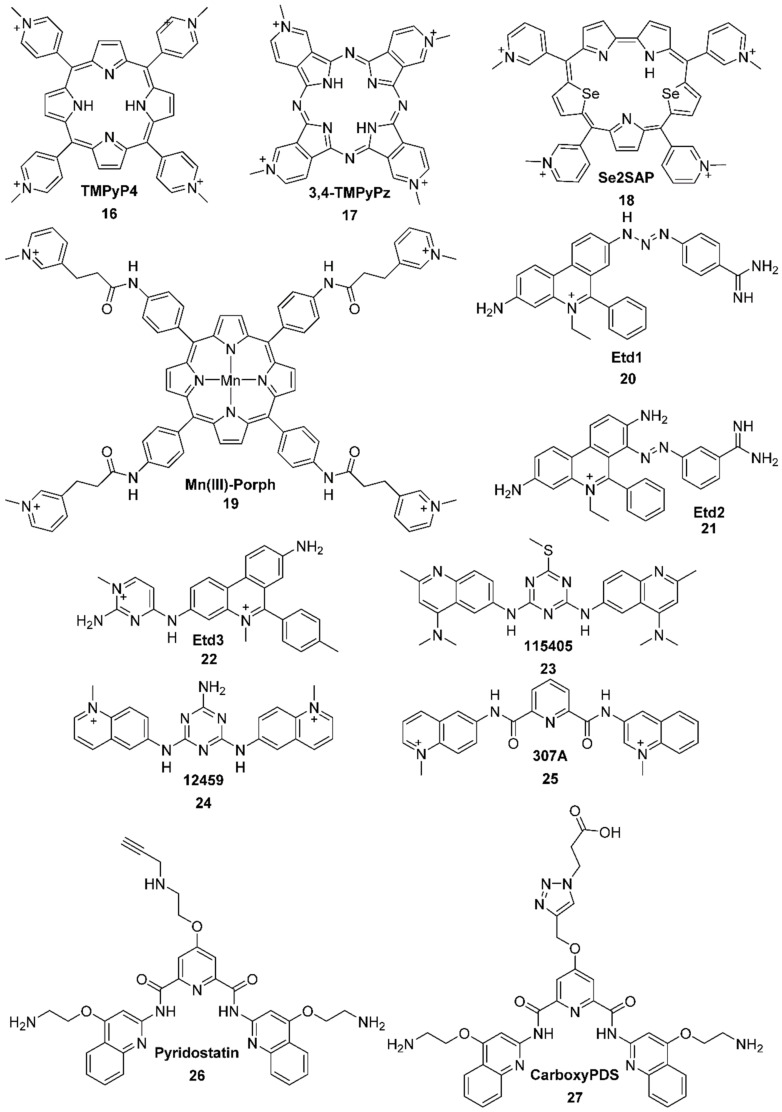
Structure of the N-methylated ligands. **16**—TMPyP4. **17**—3,4-TMPyPz. **18**—Se2SAP. **19**—Example of a Mn(III) complex bearing a porphyrin unit. **20–22**—Ethidium derivatives (Etd1, Etd2, Edt3). **23** + **24**—Triazine derivatives (115405, 12459). **25**—307A. **26**—Pyridostatin. **27**—CarboxyPDS.

Those ligands consist of a central aromatic unit with four nitrogen atoms. The nitrogen atoms located on the lateral aromatic rings are methylated, allowing easier solvation in water. Moreover, the weaker electronic density decreases the repulsion and allows better packing. TMPyP4 (Figure 6, compound **16**) [65,112,113,114,115,116,117,118,119,120] shows an inhibitive activity of telomerase (IC_50_ = 8 µM) [114] and also plays a role in the gene expression such as *c-myc*. The interaction occurs when a porphyrin unit stacks on the external quartet of the parallel G-quadruplex [121]. However, this ligand does not exhibit a much better affinity for G4 over double-stranded DNA [122,123,124,125]. Other binding geometries exist between this ligand and the G4 structures. Aside from π-stacking with the external quartet, it also can intercalate between two tetrads [121,126,127] and interact with the loops [72]. Some derivatives were studied as well. For example, 3,4-TMPyPz (Figure 6, compound **17**) exhibits a better selectivity for G4 structures, and a much higher affinity [128].

To increase the selectivity for G4 structures over double-stranded DNA, a manganese(III) center was used in the core of the ligand, composed of a porphyrin unit connecting four lateral chains bearing methylated nitrogen atoms (Figure 6, Mn(III)-Porph **19**) [129]. It was shown that the porphyrin unit lies on the top of an external tetrad while the lateral flexible cationic arms interact with the grooves [129].

The aromatic unit of porphyrins was extended with the same goal in mind. In this context, Se2SAP (Figure 6, compound **18**) shows a better selectivity for G-quadruplexes over duplexes. The studies showed that its presence induces a change of conformation from parallel or antiparallel to a hybrid one [130,131].

Most of the ligands presented so far were quite large, but some smaller ligands were also used. For example, Mergny developed some derivatives of ethidium (Figure 6, Etd1 **20**, Etd2 **21**, Etd3 **22**) [132]. However, despite a good stabilization of G-quadruplexes and the inhibition of telomerase, they were quickly replaced by derivatives of triazine (Figure 6, 115405 **23**, 12459 **24**) [133,134,135,136] because of the carcinogen risks linked to ethidium bromide. In parallel, pyridocarboxamide derivatives were also studied [137,138]. Among these compounds, one of the most promising ones in terms of G-quadruplex vs duplex selectivity and telomerase inhibition activity (IC_50_ = 0.3 µM), 307A (Figure 6, compound **25**), proves to be an efficient stabilizer of *c-myc* G-quadruplex. Furthermore, this 2,6-pyridine-dicarboxamide derivate interferes with telomere maintenance and multiple steps of the cell division cycle, namely the S-phase.

Among the other ligands that were studied, pyridostatin (PDS) (Figure 6, compound **26**) shows an effect on the growth of cancerous cells by inducing replication as well as transcription-dependent DNA damage. After 48 h of incubation at a concentration of 10 µM, pyridostatin proves to be an efficient growth inhibitor of some human cancer cell lines [139]. Another one, carboxyPDS (Figure 6, compound **27**) shows a preference for RNA quadruplexes over DNA quadruplexes [140].

#### 2.1.2. Intercalation between Guanine Tetrads

This interaction geometry is much less common. TMPyP4 intercalates between the quartet in absence of potassium cation. In the presence of the cation, the ligands preferably bind to the grooves or stack on the external quartet [121,141,142]. The experimental results are consistent with an intercalation between the tetrads [126,143]. The visible and circular dichroism (CD) spectra display the characteristic hypochromic redshift of the Soret band (420 nm) and a negative-induced CD band in the Soret region upon binding to the G-quadruplexes through intercalation. Furthermore, molecular modelling studies predicted this intercalative binding mode rather than π-stacking to either end of the tetrad.

#### 2.1.3. Interaction with Grooves and Loops

**Figure 7:**
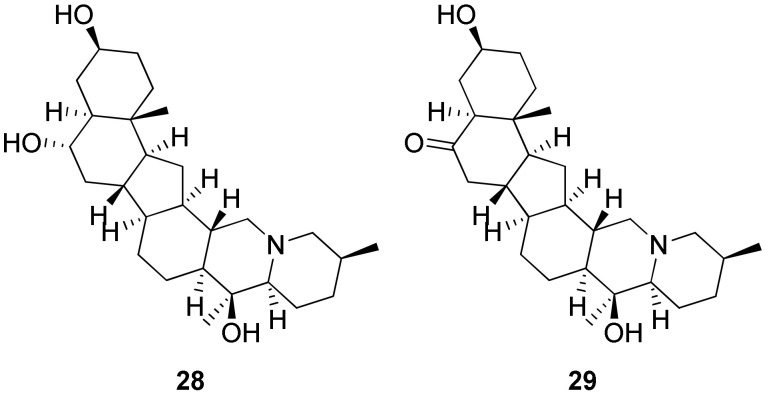
Structures of peimine **28** and peiminine **29**.

The non-planar structure of peimine et peiminine (Figure 7, **28** and **29**, respectively) confers to those ligands a higher affinity due to a better fitting into the lateral grooves of parallel G-quadruplexes by electrostatic interactions. Indeed, as the antiparallel G4 conformation, which is more prevalent in Na^+^ medium, has loops close to the grooves, these non-planar compounds display preferential binding to mixed-hybrid G-quadruplexes in K^+^ medium due to steric hindrance of the antiparallel G4 conformation adopted in Na^+^ medium. Temperature-dependent circular dichroism experiment results are in agreement with this observation and revealed selectivity over duplex DNA [144].

#### 2.1.4. Central Insertion

**Figure 8:**
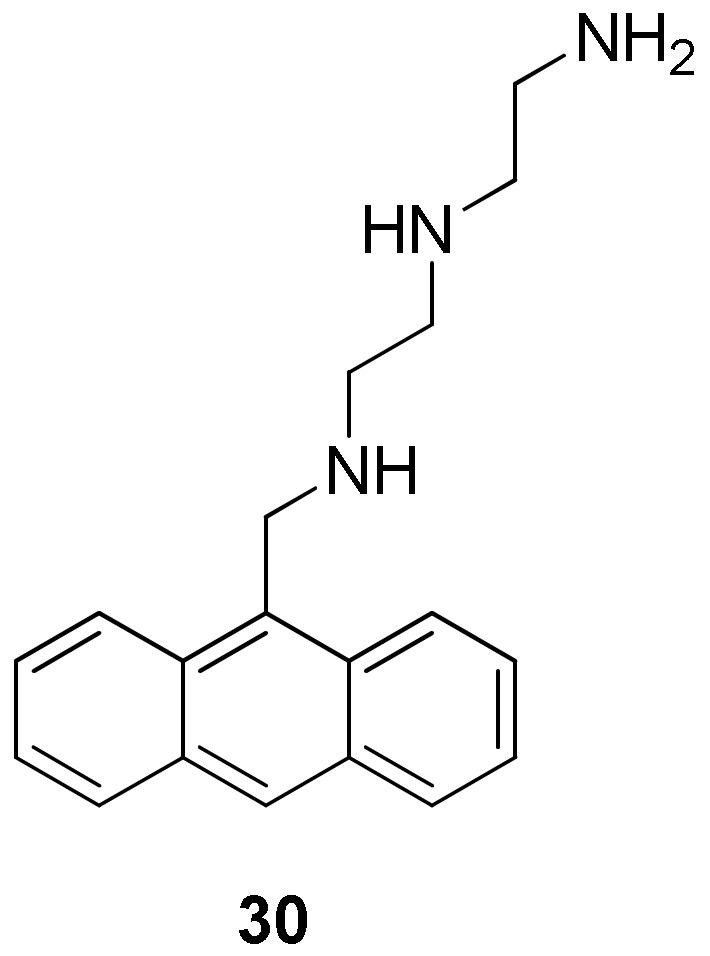
Structure of an anthracene with a polyamine chain.

Since the guanines’ carbonyl groups face each other at the center of the quartet, the high electronic density attracts small cations such as K^+^ and Na^+^, which place themselves in the central channel. In this context, some ligands were designed with an aromatic part that interacts with the external quartet while a polyamine chain penetrates inside the central channel (Figure 8, **30**) [145].

### 2.2. Metal Complexes-Based G4 Ligands

Metal complexes display interesting photophysical properties that can be exploited to target G-quadruplexes. For instance, such complexes can emit light specifically when bound to G4 structures [146] or stabilize a telomeric G4 conformation [147].

Moreover, the presence of a metallic ion near the central channel can optimize the alignment of the aromatic moieties of the ligands and the guanines, resulting in a stronger interaction force [148].

#### 2.2.1. Porphyrines

**Figure 9:**
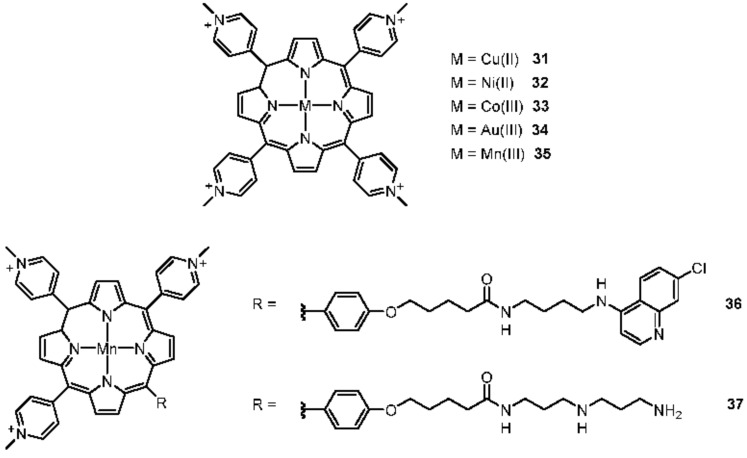
Structure of porphyrin ligands used with various metal ions for G-quadruplexes recognition.

As explained before, TMPyP4 has been widely studied and metal complexes have been designed as well, leading to the synthesis of complexes with various metal cations: Cu(II) [149,150], Ni(II), Co(III), Au(III), and Mn(III) (Figure 9, **31**–**35**) [151].

Other Mn(III)-porphyrin complexes (Figure 9, **36** and **37**) were imagined to specifically target G-quadruplexes. For this purpose, the porphyrin unit stacks on the external tetrads while lateral arms interact with the grooves [129,152,153,154]. The resulting Mn(III)-porphyrin complexes display a lower inhibitory activity of telomerase than TMPyP4, but remain efficient G-quadruplex stabilizers. Other porphyrin complexes built around Zn(II) [128,155] have been studied such as the 3,4-TMPyPz zinc(II) porphyrazine [128]. By adding a metal center, the binding affinity towards G-quadruplexes was weakened compared to the 3,4-TMPyPz parent ligand, despite the four molecules of 3,4-TMyPyPz zinc(II) that can bind to telomeric G-quadruplex. Among other porphyrin, a protoporphyrin IX complex incorporating a Fe(III) cation, also known as haemin, has been widely discussed and displays a strong binding affinity of 4.2 × 10^7^ M^−1^ [156,157].

#### 2.2.2. Salphens

**Figure 10:**
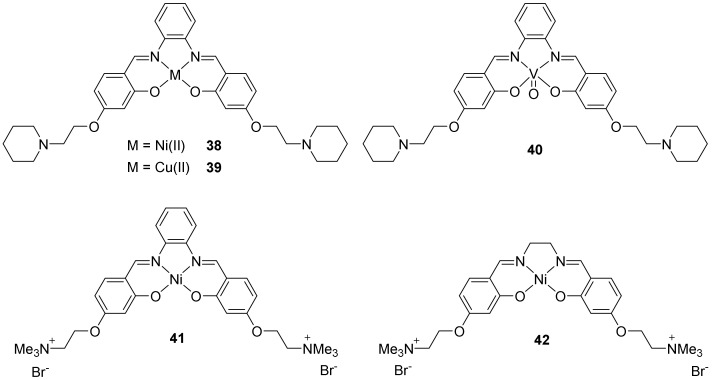
Structure of metalled salphen and salen complexes used for G-quadruplexes recognition.

Another category of metal complexes is based on a salphen unit. Cu(II) and Ni(II)-salphen complexes (Figure 10, **38**, **39**) have been synthetized in this framework [148,158] and are good stabilizers of G4-structures in consequence of the stacking of the salphen rings on the external tetrads and the interactions of the lateral charged substituents with the grooves. The metal center is located above the central channel [159]. While complexes adopting a square planar geometry develop a good affinity for G4 quadruplexes, as expected, it was demonstrated that complexes with a square-based pyramidal geometry also develop a good affinity for G-quadruplex in addition to a good selectivity for those structures compared to duplex DNA. The vanadyl complex presented in Figure 10, **40** is a representative example of this family of salphen square-based pyramidal compounds [158].

Based on this concept, other metals were used and various complexes were developed: Ni(II), Pd(II), Cu(II), Co(III), Pt(II), or Zn(II) [160,161,162,163,164,165,166,167,168,169]. Most of these salphen or salen complexes display a square planar geometry except the square-based pyramidal salphen complexes based on Co(III) [168].

A study compared two Ni(II) complexes bearing a salphen (**41**) or a salen (**42**) ligand (Figure 10) [170]. The presence of that supplementary aromatic ring increases the interaction by π-stacking. The presence of cationic groups on the lateral chains increases the affinity [170], whereas anionic groups decrease it [171].

#### 2.2.3. Terpyridines and Other Square Planar Complexes

**Figure 11:**
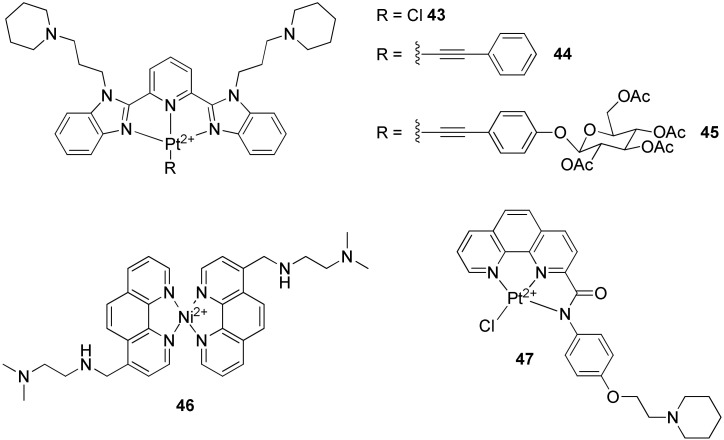
Structure of some terpyridines and other square planar complexes used for G-quadruplex recognition.

Square planar geometry adopted by Pt(II) and Pd(II) complexes wearing terpyridine ligands has been proved to be necessary for a good affinity when compared with Cu(II) and Zn(II) analogue complexes which are not planar [172]. The interaction geometry remains based on stacking on top of the external tetrads, but some studies demonstrated that interactions within the loops were also possible [173,174,175]. The terpyridine geometry was also modified to increase the affinity towards G-quadruplexes structures. Pt-bzimpy [176] (Figure 11, **43**–**45**) is a series of compounds synthesized for this purpose.

Another example is (K34)_2_Ni(II) (Figure 11, **46**) which has a high affinity to the external tetrads thanks to its square planar geometry [177]. Another square planar Pt(II) complex based on a mono-substituted phenanthroline ligand (Figure 11, **47**) shows a good stabilization of G-quadruplexes by stacking on top of G-tetrads [178]. Molecular calculations also predicted the fitting of the piperidine sidearm into the pocket created by a TTA loop. Furthermore, this compound presents a 40-fold selectivity for G-quadruplexes over duplex DNA and is, therefore, more selective than BRACO-19. However, its telomerase inhibitory activity is less effective than BRACO-19 or TMPyP4, with an IC_50_ value of 49.5 µM (compared to 2.5 µM [84] and 8 µM [114], respectively).

Besides those metal complexes adopting mainly a square planar geometry, complexes adopting an octahedral geometry have also been studied with G-quadruplexes as for example complexes based on Fe(III) [179] or Ir(III) [180,181,182,183]. Among those complexes, the ruthenium(II) complexes occupy a central place and will be at the core of the following sections.

## 3. Interactions of Ruthenium(II) Complexes with G-quadruplexes

**Figure 12:**
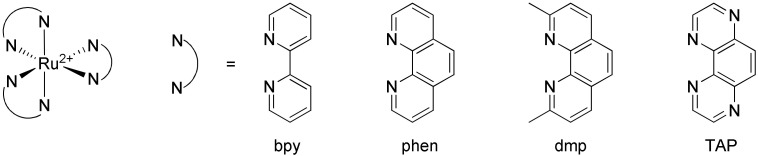
Schematic structure of ruthenium(II) polypyridyl complexes and of typical ancillary ligands.

Ruthenium(II) polypyridyl complexes are composed of one central ruthenium(II) cation and three chelating diimines. The first example of the luminescence of such complexes dates from the report of the luminescence of [Ru(bpy)_3_]^2+^ (bpy = 2,2′-bipyridine) by Paris and Brandt [184], who paved the way for an impressive field of research. This luminescence arises from a metal-to-ligand charge transfer triplet excited state (^3^MLCT) whose energy largely depends on the solvent used. The photophysical and electrochemical properties of Ru(II) polypyridyl complexes can be finely tuned by the structure of the chelating diimines which allowed their use in a plethora of applications involving light-induced processes [185,186]. A lot of these complexes are composed of one ligand of interest and two other “spectator” ancillary ligands which play an important structural role but do not control the photophysical or electrochemical properties of the complex. The best examples of ancillary ligands are the bpy and phen (1,10-phenanthroline) molecules and derivatives thereof such as the dmp (2,9-dimethyl-1,10-phenanthroline) or TAP (1,4,5,8-tetraazaphenanthrene) molecules (Figure 12) [187].

**Figure 13:**
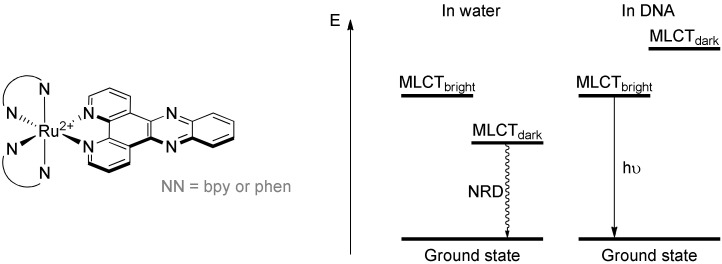
Structure of [Ru(L)_2_(**dppz**)]^2+^ (L = bpy or phen) and energy diagram of the excited states involved in the light-switch effect in function of the environment around the complex. NRD = non-radiative decay.

The most famous compounds triggering the interest of ruthenium(II) polypyridyl complexes as powerful tools to study DNA are the [Ru(bpy)_2_(**dppz**)]^2+^ and [Ru(phen)_2_(**dppz**)]^2+^ (dppz = dipyrido[3,2-*a*:2′,3′-*c*]phenazine) complexes [188]. These complexes are called light-up probes as they are not luminescent in water but do emit brightly in DNA containing aqueous solutions. This light-switch ON effect can be explained by the presence of two different MLCT excited states whose relative energy position is very sensitive to the environment: one bright emissive state and one dark non-emissive state [189,190]. In water, the formation of hydrogen bonds with the nitrogen atoms of the pyrazinic cycle greatly stabilizes the dark state, which leads to a very strong luminescence quenching as no luminescence can be observed anymore. However, in apolar solvents or in presence of DNA, no hydrogen bonds are formed and the lowest excited state is the bright state, thus restoring the luminescence of the complex (Figure 13).

The first studies involving ruthenium(II) complexes and G-quadruplexes started with [Ru(bpy)_3_]^2+^ as Szalai and Thorp compared its cyclic voltammogram in the presence of G4s relative to the results obtained with duplex DNA (B-DNA) [191]. Their work revealed that guanines of G-quartets are more accessible to the complex than guanines of duplexes. However, the three-dimensional structure of G4s does not allow for sufficiently compact stacking to constitute an increase in the reactivity observed for sequences with successive guanines in duplex DNA.

On top of that, other very well-known homoleptic ruthenium(II) complexes, namely [Ru(phen)_3_]^2+^ and [Ru(TAP)_3_]^2+^ (TAP = 1,4,5,8-tetraazaphenanthrene), were studied in the presence of G4 structures as well [192]. These complexes only interact weakly with G-quadruplexes. However, it appears that [Ru(phen)_3_]^2+^ interacts through different binding modes, namely by end-stacking and intercalation into the minor groove, which can induce slight deformation in the G-quadruplex structures.

The following section will focus on the interaction of ruthenium(II) polypyridyl complexes with G-quadruplexes starting with mononuclear and then polynuclear complexes. The main focuses of this section are first dppz containing complexes as their interaction with G4s are the most understood due to the advanced techniques used for their study. Afterwards, ruthenium(II) complexes containing a π-extended ligand will be thoroughly explored as large aromatic surfaces have been demonstrated to promote G-quadruplexes selectivity through improved interaction of the complexes with these structures.

The last section of this review will be discussing the photoreactivity of certain types of ruthenium(II) polypyridyl complexes with G-quadruplexes for their irreversible labelling.

### 3.1. Monometallic Ruthenium(II) Complexes

#### 3.1.1. Ruthenium(II) dppz and dppz Derivative Complexes

The first examples of the use of [Ru(L)_2_(**dppz**)]^2+^ (L = bpy or phen) as sensor for G-quadruplexes dates from 2010 with two studies carried out by Shi et al. [193,194]. Both complexes show an increase in luminescence when interacting with either G-quadruplexes or i-motif DNA. However, the increase is more intense with G-quadruplexes indicating a better protection of the complex from this type of structure and thus better interaction. Furthermore, the increase in luminescence is more important with mixed-hybrid G-quadruplexes which are more prevalent in K^+^ medium, whereas the luminescence increase is lower with antiparallel G-quadruplexes which are more present in Na^+^ medium. This indicates a further potential selectivity of such complexes towards specific structures of G-quadruplexes. These first studies showed promising results and paved the way for further understanding of the interaction of these complexes with G-quadruplexes and the consequent impact on the photophysical behavior of dppz containing Ru(II) complexes.

As both DNA and the studied complexes are chiral, the following studies explored the possible differences of the interaction of Δ and Λ enantiomers of [Ru(L)_2_(**dppz**)]^+^ with G-quadruplexes [195,196]. Interestingly, the Λ enantiomer of both complexes show a slightly stronger binding than the Δ enantiomer, which is contrary to the observations reported when such complexes interact with double-stranded DNA instead of G-quadruplexes. On the other hand, the chirality of the complexes does not have an impact on the affinity of the complex towards different structures of G-quadruplexes (hybrid or antiparallel) indicating the absence of appreciable stereoselective G-quadruplex binding.

**Figure 14:**
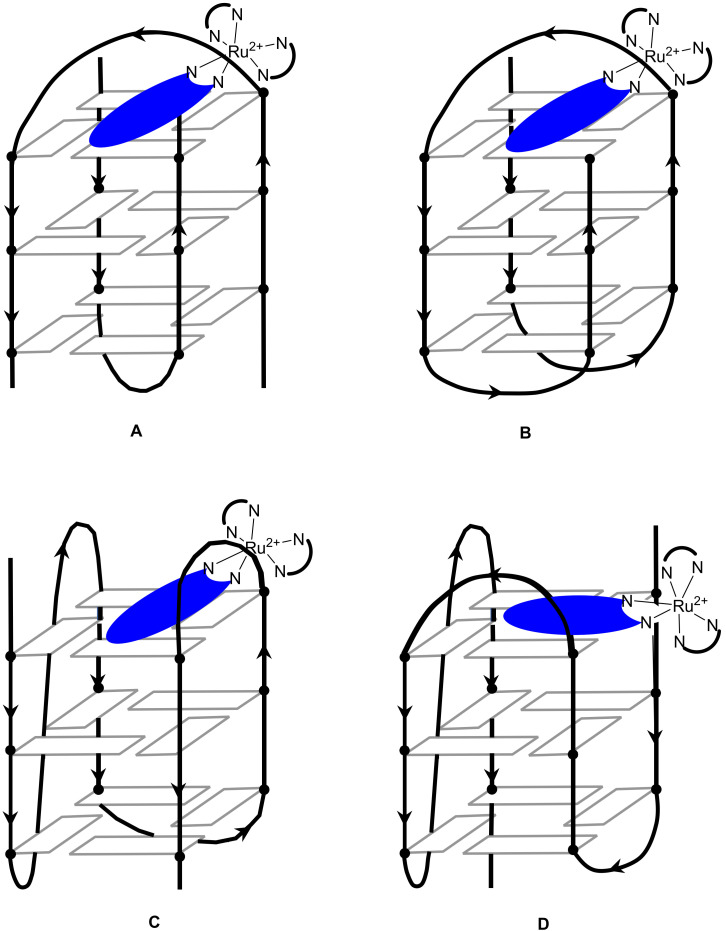
Binding interactions of [Ru(phen)_2_dppz]^2+^ with (**A**)—G4T4G4 (K^+^), (**B**)—G-HT (Na^+^), (**C**)—G-HT (K^+^) Hybrid 1, (**D**)—G-HT (K^+^) Hybrid 2; using time-resolved infrared spectroscopy as proposed by Devereux et al. [197] (G4T4G4 = *Oxytricho nova* telomere sequence [d(G_4_T_4_G_4_)]_2_, G-HT = human telomere sequence d[AG_3_(T_2_AG_3_)_3_]).

Using ultrafast time-resolved infrared spectroscopy allowed a recent investigation on the binding site of [Ru(phen)_2_(**dppz**)]^2+^ with various G-quadruplex structures in solution [197]. This technique allows one to get information on both the excited state of the complex (bright or dark state) and on the influence of the dipole of the excited complex on the interacting DNA bases. As expected, different structures of G-quadruplexes induce a different geometry of interaction with the striking highlighting of the thymine base interactions in the binding of the complex with the Na^+^-stabilized antiparallel human telomer quadruplex (Figure 14B). This indicates that instead of stacking on to the terminal guanine quartets, [Ru(phen)_2_(**dppz**)]^2+^ seems to bind in the loop region of the antiparallel G-quadruplex structure. However, for the other structures studied, signals attributed to the interaction of the complex with guanines indicates that the complex is stacked on the terminal guanine quartets with varying degrees of interaction with other DNA bases depending on the size and flexibility of the loops (Figure 14A,C,D).

Even more recently, the ultrafast excited state dynamics of [Ru(phen)_2_(**dppz**)]^2+^ was studied in order to investigate the influence of the microenvironment around the complex on the population of both the bright and dark states [198]. The ultrafast dark state formation and decay rates are the most sensitive to the amount of water near the complex. The faster formation of the dark state (10.5 ps) for [Ru(phen)_2_(**dppz**)]^2+^ interacting with the antiparallel Na^+^-stabilized human telomeric sequence when compared to the 15.6 ps obtained in presence of *Oxyticha nova* G-quadruplex sequence indicates that the microenvironment of the complex is more hydrophilic for the former interaction site. This finding agrees with the previously discussed study which pointed out that the complex interacts with the loops of this antiparallel Na^+^-stabilized structure of G-quadruplex. Such loops allow a faster water exchange since they offer a more flexible environment for the complex.

**Figure 15:**
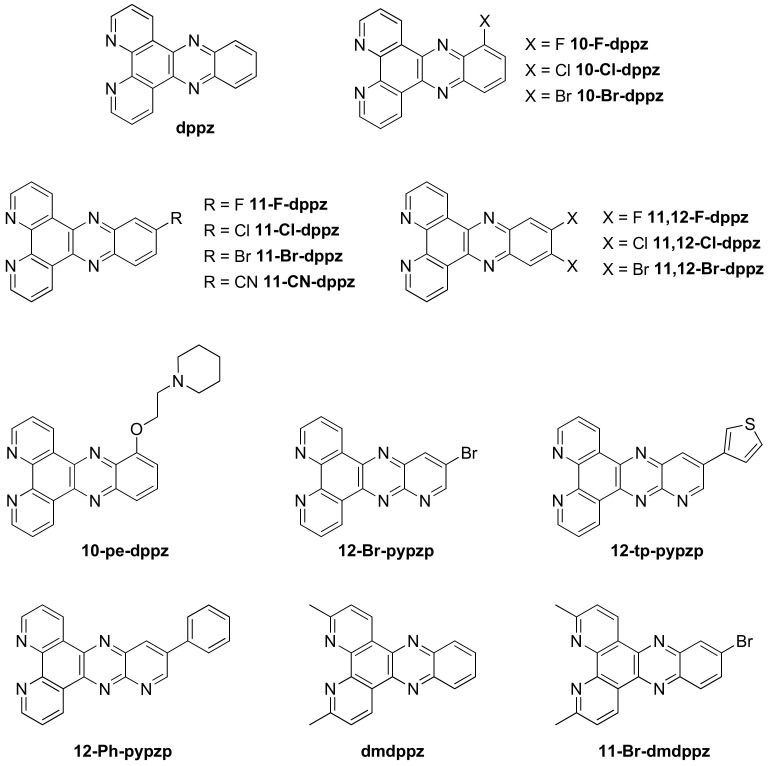
Structure of dppz and closely related ligands used to form ruthenium(II) complexes studied in presence of G-quadruplexes.

These recent studies underscore the ability to potentially discriminate different secondary structures of DNA with such complexes and thus emphasize the need to understand their behavior in a precise way. Furthermore, the ability to greatly modify the structure of the dppz-type ligands (Figure 15) opens the way to further development in the quest for even more selective probes which are discussed here below.

**Figure 16:**
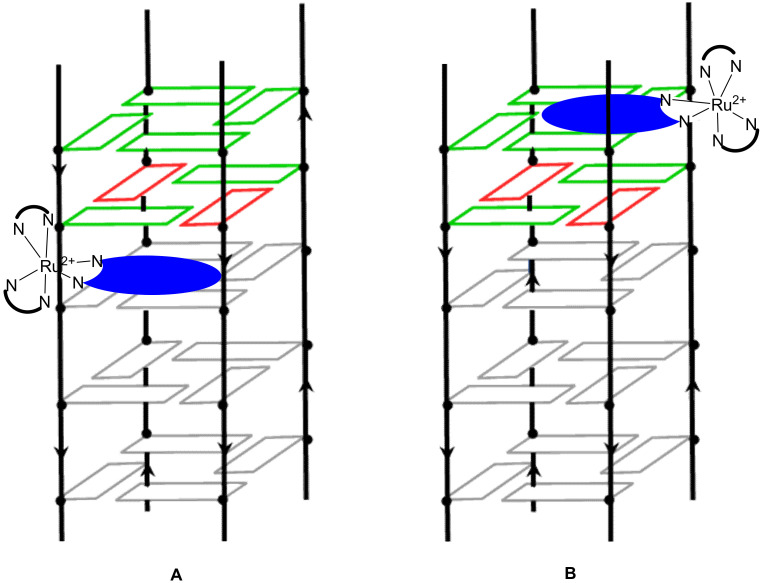
Binding interactions of (**A**)—Λ-[Ru(TAP)_2_(11-CN-dppz]^2+^ and (**B**)—Λ-[Ru(phen)_2_(11-CN-dppz]^2+^ with parallel tetramolecular G-quadruplex d(TAGGGTTA) using X-ray crystallography as proposed by McQuaid et al. [200] where adenine, guanine and thymine are colored in red, grey, and green, respectively and the 11-CN-dppz ligand is colored in blue.

Obtaining a crystal structure of a complex interacting with a G-quadruplex is rather challenging due to the various structures they can adopt. However, in 2019 two reports were published on the crystal structure of Λ-[Ru(TAP)_2_(**dppz**)]^2+^ [199] and the closely related Λ-[Ru(TAP)_2_(**11-CN-dppz**)]^2+^ as well as Δ-[Ru(phen)_2_(**11-CN-dppz**)]^2+^ (Figure 15) [200] bound with the G-quadruplex forming heptamer d(TAGGGTT). Out of the four binding modes found for Λ-[Ru(TAP)_2_(**dppz**)]^2+^, none of them show the complex stacking on one of the ends of the G-quadruplex. Indeed, the four structures obtained show that the complex is rather interacting with the thymine–adenine loop regions, which agrees with the recent spectroscopic findings in solution. The different binding sites observed might also explain the various degrees of protection from water of the complex and thus the various lifetimes measured in solution. Adding a simple cyano group on the dppz ligand to form [Ru(TAP)_2_(**11-CN-dppz**)]^2+^ drastically changes the obtained crystal structure. Indeed, the Λ enantiomer is shown to interact directly with the G-tetrad plane (Figure 16A), which is not the case for [Ru(TAP)_2_(**dppz**)]^2+^. On the contrary, Δ-[Ru(phen)_2_(**11-CN-dppz**)]^2+^ does not interact directly with the G-quartet but rather with the terminal T-T pairs (Figure 16B). These findings further illustrate the need to use advanced techniques to better understand the implications of small structural changes of the complexes on their ability to interact with G-quadruplexes.

A few articles illustrate the impact of small structural changes on the binding affinity and sensing ability of various complexes in presence of various structures of G-quadruplexes [201,202,203,204,205]. However, despite showing drastic differences in the emission profile of the complexes in function of their structure and the type of G-quadruplexes they bind to, the lack of systematic study with thorough structural investigation does not allow the establishment of a systematic structure–activity relationship.

**Figure 17:**
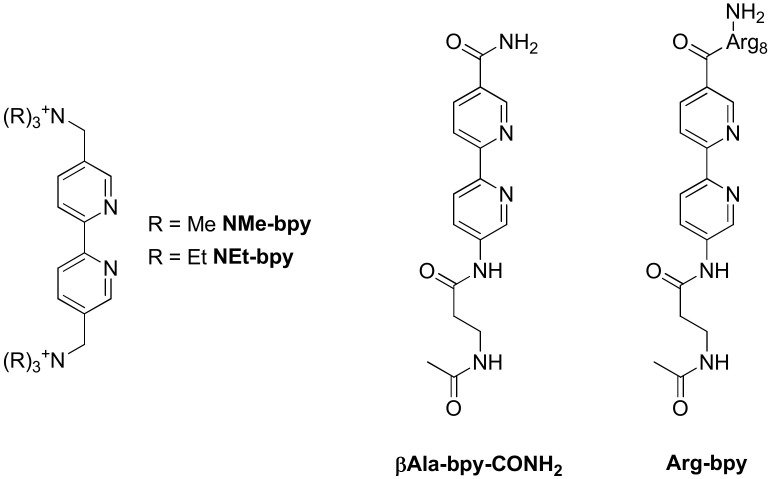
Structure of ancillary ligands used with dppz-containing ruthenium(II) complexes.

Some complexes containing two dppz ligands have also been investigated ([Ru(**NMe-bpy**)(**dppz**)_2_]^4+^, [Ru(**NEt-bpy**)(**dppz**)_2_]^4+^, [Ru(**βAla-bpy-CONH_2_**)(**dppz**)_2_]^2+^ and [Ru(**Arg-bpy**)(**dppz**)_2_]^2+^) (Figure 17) [206,207] but no drastic differences other than a slightly higher affinity to the G-quadruplexes can be noticed from the complexes containing only a single dppz ligand. However, the introduction of a peptide sequence (L-octaarginine) on the complex changes its selectivity between different G-quadruplex structures.

Incorporating methyl groups at positions 3 and 6 of the dppz ligand alters the well-studied [Ru(bpy)_2_(**dppz**)]^2+^ into a dual photochemical “light-switch” and DNA damaging agent [208]. The new complex, [Ru(bpy)_2_(**dmdppz**)]^2+^ (dmdppz = 3,6-dimethyl-dipyridophenazine) (Figure 15), undergoes photo-induced ligand ejection in organic solvents and upon interaction with duplex and G-quadruplex DNA, consequently generating a ligand-deficient and reactive metal center which then leads to covalent metalation of DNA. The photo-ejection is confirmed by a redshift in the absorption spectra and decreased extinction coefficient, with a selective ligand ejection since only the dmdppz ligand is ejected.

A small structural change by adding a bromine atom at position 11 (**11-Br-dmdppz**) (Figure 15) enhances the G-quadruplex selectivity while maintaining the ligand ejection phenomenon [205]. However, these strained complexes, [Ru(bpy)_2_(**dmdppz**)]^2+^ and [Ru(bpy)_2_(**11-Br-dmdppz**)]^2+^, are more likely to interact with one side of hybrid G-quadruplex structures by binding through an end-capping mode on the lateral loop end due to the distorted planar dppz ligand.

Another structural change on the dppz ligand by incorporating a flexible chain generates a new complex [Ru(bpy)_2_(**10-pe-dppz**)]^2+^ (10-pe-dppz = 10-(2-(piperidin-1-yl)ethoxy)dipyrido[3,2-*a*:2′,3′-*c*]phenazine) (Figure 15), displaying a great ability to stabilize the G-quadruplex conformation in the K^+^ medium by interacting through the stacking mode on the G-tetrads [209]. Further studies, through polymerase chain reaction (PCR) stop and telomerase repeat amplification protocol (TRAP) assays, show the potential use of this ruthenium(II) complex as a telomerase inhibitor with no acute cytotoxicity at the same time with an IC_50_ value of 38.8 µM. 

#### 3.1.2. Ruthenium(II) Complexes Bearing a π-Extended Ligand

**Figure 18:**
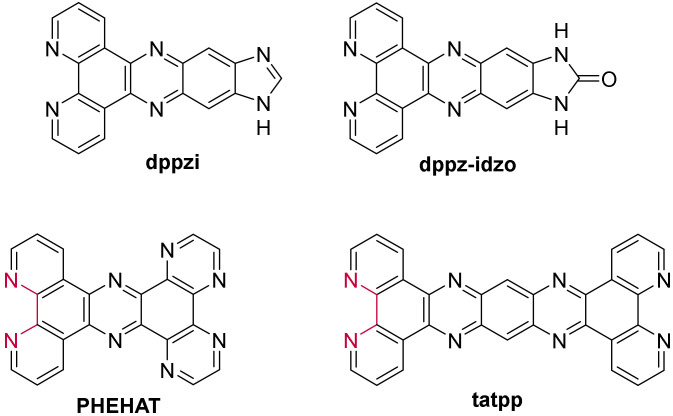
Structure of the π-extended symmetrical ligands form ruthenium(II) complexes studied in presence of G-quadruplexes.

In view of designing new ruthenium(II) complexes as probes for G-quadruplexes, researchers started extending the conjugated system of the aforementioned dppz ligand. A series of complexes containing the **dppzi** (= dipyrido[3,2-*a*:2′,3′-*c*]phenazine-10,11-imidazole) (Figure 18) ligand were therefore reported by Shi et al. [210,211]. Similar to [Ru(phen)_2_(**dppz**)]^2+^, [Ru(bpy)_2_(**dppzi**)]^2+^ shows a preferred interaction with G-quadruplexes relative to i-motif. Additionally, this complex binds preferentially to the mixed-hybrid G-quadruplex rather than the antiparallel basket-type conformation, which is consistent with the higher stabilizing effect observed in the K^+^ buffer [210]. The greater binding affinity towards the mixed-hybrid G-quadruplex over that towards antiparallel G-quadruplex seems to follow the same trend as what was observed with the other studied [Ru(L)_2_(**dppzi**)]^2+^ complexes (L stands either for phen or dmp = 2,9-dimethyl-1,10-phenanthroline) [196]. Further studies revealed the effect of the ancillary ligands on the spectral properties of the complex upon interaction with G-quadruplexes. [Ru(phen)_2_(**dppzi**)]^2+^ is emissive in the absence and presence of G-quadruplexes, whereas adding the -CH_3_ substituent on the ancillary phen ligands results in no luminescence at all for [Ru(dmp)_2_(**dppzi**)]^2+^, regardless of the considered experimental conditions [196]. Surprisingly, however, [Ru(bpy)_2_(**dppzi**)]^2+^ displays a so-called reversible “light-switch” behavior that can be regulated by the competition of [Fe(CN)_6_]^4−^ ions and G-quadruplex DNA. In fact, upon the addition of G-quadruplexes, the emission intensity of [Ru(bpy)_2_(**dppzi**)]^2+^ is enhanced by about 2.5 while the luminescence is completely quenched upon the addition of [Fe(CN)_6_]^4−^ ions in the absence of G-quadruplex DNA. The luminescence can be restored by adding an excess of G-quadruplexes to the system containing both the G4-bound complex and [Fe(CN)_6_]^4−^ ions [210].

On the contrary, the ancillary ligands do not have any effect regarding the binding modes of each complex with G4-quadruplexes, namely stacking on the center between the parallel loop and the terminal G-quartet in Na^+^ buffer and π-stacking on the terminal G-quartets in K^+^ buffer. By adding the -CH_3_ substituents, the resulting complex presents more steric hindrance preventing [Ru(dmp)_2_(**dppzi**)]^2+^ from interacting less efficient with G-quadruplex DNA than the bpy- and phen-analogue complexes [210,211].

As these complexes do not display high emission enhancement upon binding to G-quadruplex DNA, further small structural changes were made by introducing an imidazolone group to the dppz ligand. A noteworthy emission intensity enhancement was observed upon the addition of G-quadruplexes to the [Ru(L)_2_(**dppz-idzo**)]^2+^ complexes (L = bpy or phen; **dppz-idzo** = dppz-imidazolone (Figure 18)) in K^+^ medium [212,213]. Interestingly though, [Ru(phen)_2_(**dppz-idzo**)]^2+^ displays a pH-controlled light-switch effect while being in the presence of G-quadruplexes. In the acidic pH region below 1.4, the complex shows no luminescence, whereas the addition of OH^-^ ions and adjusting the pH to 4.5 restores the emission [212]. This reversible light-switch effect can be explained by the dissociation of the G-quadruplex to random-coil single-stranded DNA (ssDNA) at pH 1.4, the complex being not bound anymore to this random-coil structure and thus not protected from a water environment.

Further studies explored the binding behaviors of the Δ and Λ enantiomers of [Ru(bpy)_2_(**dppz-idzo**)]^2+^ with G-quadruplexes [195]. Reminiscent of the parent Λ-[Ru(bpy)_2_(**dppz**)]^2+^ enantiomer, Λ-[Ru(bpy)_2_(**dppz-idzo**)]^2+^ shows a stronger binding affinity with antiparallel G-quadruplex than the Δ enantiomer, which marks a great difference compared to double-stranded DNA binding. However, no chiral selectivity towards hybrid or antiparallel G-quadruplex structures was observed for the Δ and Λ isomers. Both isomers bind to G-quadruplexes by π-π stacking interactions, with a supplementary binding mode through groove binding for the Λ isomer.

More recently, Shi et al. investigated the influence of multiple factors, such as tail and loop length, linkers, and ionic concentration, on the enantioselectivity of these isomers towards the G-quadruplex structures [214]. As expected, cation concentration (K^+^ or Na^+^) influences the enantioselectivity, which is the most notable at a low concentration. Nonetheless, no remarkable chiral selectivity was observed otherwise except that the initial enantioselectivity disappears or weakens with the increasing length of TTA loops.

Simultaneously, complexes bearing an even more extended conjugated system were investigated [192]. Moucheron et al. focused their interest on a Ru(II) compound containing the planar DNA intercalating **PHEHAT** (=1,10-phenanthrolino[5,6-*b*]1,4,5,8,9,12-hexaatriphenylene) ligand (Figure 18). In the presence of G-quadruplexes, this complex also displays “light-switch ON” properties and concomitantly induces a high stabilization of G4s, with a preference towards the hybrid conformation. Furthermore, under these conditions, [Ru(phen)_2_(**PHEHAT**)]^2+^ shows a large selectivity towards G4 structures over duplex structures, as confirmed by competitive experiments. Molecular modelling of the binding of [Ru(phen)_2_(**PHEHAT**)]^2+^ to hybrid G4s gave further insight into the G4 vs duplex selectivity, showing that the G4 selectivity is due to the interaction with loops as well as the π-interaction between the adenine bases and the PHEHAT ligand [192].

Besides a high affinity of 10^6^ M^−1^, no further systematic study was conducted for [Ru(phen)_2_(**tatpp**)]^2+^ (tatpp = tetraazapyridopentacene) (Figure 18) bearing an even larger intercalative ligand than PHEHAT to provide fundamental insights into the interaction of the ruthenium(II) complex with the G4 scaffold [215].

While the foregoing ruthenium(II) polypyridyl complexes carried symmetrical planar extended ligands, the following paragraphs will be discussing the interaction of ruthenium compounds based on an elbow-shaped or non-symmetrical planar ligand with G-quadruplexes. These compounds were designed to improve the G4 vs duplex selectivity since most of the reported G-quadruplex ligands bind by π-stacking on the external G-quartets, whereas only a few can interact with grooves/loops and G-quartets simultaneously.

**Figure 19:**
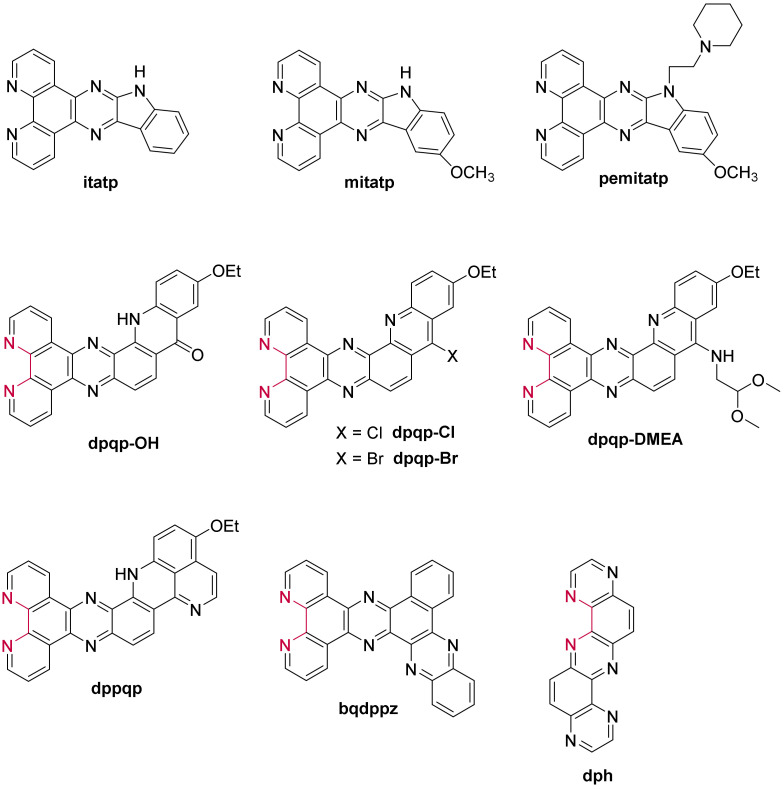
Structure of the π-extended non-symmetrical ligands to form ruthenium(II) complexes studied in presence of G-quadruplexes.

Further structural changes were consequently made on the [Ru(L)_2_(**dppz**)]^2+^ compounds by incorporating an indoloquinoline moiety [147,216], part of the alkaloid group, already discussed and reported as efficient G-quadruplex ligands. The resulting complexes efficiently stabilize the G-quadruplex structures and show moderate (~10^5^ M^−1^) [147] to high (~10^7^ M^−1^) [216] binding constants through binding by the characteristic π-π stacking interaction. Interestingly, [Ru(phen)_2_(**mitatp**)]^2+^ (**mitatp** = 5-methoxy-isatino[1,2-*b*]-1,4,8,9-tetraazaphenylene) (Figure 19) can induce the formation of a parallel G-quadruplex structure [147], hitherto rarely reported, as most ruthenium(II) polypyridyl complexes convert the human telomeric sequence into an antiparallel or hybrid conformation. On another note, the absence of a methoxy group generates a complex with a higher binding constant and provides the ability to discriminate between different G-quadruplex structures [216].

Liao et al. eventually incorporated a flexible chain on the mitatp ligand to design a new complex, namely [Ru(bpy)_2_(**pemitatp**)]^2+^ (**pemitatp** = 5-methoxy-1-(2-(piperidin-1-yl)ethyl)-isatino[1,2-*b*]-1,4,8,9-tetraazatriphenylene) (Figure 19), capable of effectively affecting the activity of the telomerase and stabilizing G-quadruplexes [209]. Similar to the structurally similar complex [Ru(bpy)_2_(**10-pe-dppz**)]^2+^, this compound shows no acute cytotoxicity at concentrations up to 3 µM, with an IC_50_ of 39.7 µM.

To improve the G4 binding ability and selectivity to a greater extent, the Moucheron and Demeunynck groups designed a new series of complexes with a π-extended acridine-derived ligand (Figure 19) [217,218]. The G4 interaction of those elbow-shaped ruthenium(II) complexes was assessed by fluorescence resonance energy transfer (FRET)-melting assays against an array of quadruplex-forming DNA and RNA sequences and a duplex DNA sequence. These studies revealed that the complexes stabilize both DNA and RNA G-quadruplexes. Furthermore, they can discriminate between quadruplex and duplex sequences as demonstrated by competitive experiments. Their selectivity towards G-quadruplexes over duplexes is partly attributed to the acridine moiety of the π-extended ligands [218], thus allowing more favorable π-stacking interactions between the ligand and the G-quadruplexes, already reported by Neidle [219,220]. Further FRET-melting experiments showed that beyond the interaction with the external quartets, these complexes also interact with the loops [218].

Additionally, the complexes bearing a halide substituent on the acridine moiety, [Ru(phen)_2_(**dpqp-Cl**)]^2+^ and [Ru(bpy)_2_(**dpqp-Br**)]^2+^ (dpqp = dipyrido[3,2-*a*:2′,3′-*c*]quinoli- no[3,2-*h*]phenazine), display remarkable spectroscopic properties upon interaction with nucleic acids, which make them very interesting light-up probes. They show little to no luminescence at all in aqueous solution and moderate luminescence with duplexes, while there’s a remarkable luminescence enhancement (up to 330) in the presence of G-quadruplexes. The combined studies also showed that they are efficient at labelling G-quadruplex structures even against an excess of duplex DNA in two orders of magnitude higher concentrations. Following the particular light-up properties of the acridine derived ruthenium(II) complexes, the most promising complex, [Ru(bpy)_2_(**dpqp-Br**)]^2+^ was further assessed through preliminary cellular luminescent labelling studies by incubation of melanoma B16F10 cells [218]. Confocal images of the cancer cells gave insights into the main labelling sites, the nucleoli, and diffuse perinuclear cytoplasmic foci. These results are evocative of previously reported quadruplex-selective smart probes [221,222], suggesting that the complex primarily labels RNA G-quadruplexes, rooted in the nucleolus and cytoplasmic assemblies of ribonucleoprotein particles [218].

Another ruthenium complex based on the non-symmetrical ligand **bqdppz** (= benzo[*j*]quinoxalino[2,3-*h*]dipyrido[3,2-*a*:2′,3′-*c*]phenazine) (Figure 19) [223,224] was investigated and revealed a remarkable luminescent enhancement towards hybrid G-quadruplex as opposed to the antiparallel structure. Due to the steric hindrance generated by the loops, end-stacking on hybrid G-quadruplexes is more favorable, which allows a preferential stabilization of this hybrid structure.

Recently, Elias et al. designed a ruthenium(II) complex incorporating a new planar-extended ligand, based on a pyrazino core, the **dph** (= dipyrazino[2,3-*a*:2′,3′-*h*]phenazine) (Figure 19) [225]. A combined analysis of the spectroscopic properties, computational data, and Surface Plasmon Resonance (SPR) experiments showed that this complex exhibits a higher affinity and specificity towards G-quadruplexes than double-stranded DNA (4.8 × 10^4^ M^−1^ vs 1.3 × 10^3^ M^−1^). Despite displaying two binding modes, mainly the π-stacking interaction between the dph ligand and guanines from the G-quartet with minor contribution by insertion into the groove, the binding affinity for G-quadruplexes remains lower than the previously reported ruthenium(II) complexes.

#### 3.1.3. Ruthenium(II) Complexes Bearing Imidazo-Phenanthroline Ligands

**Figure 20:**
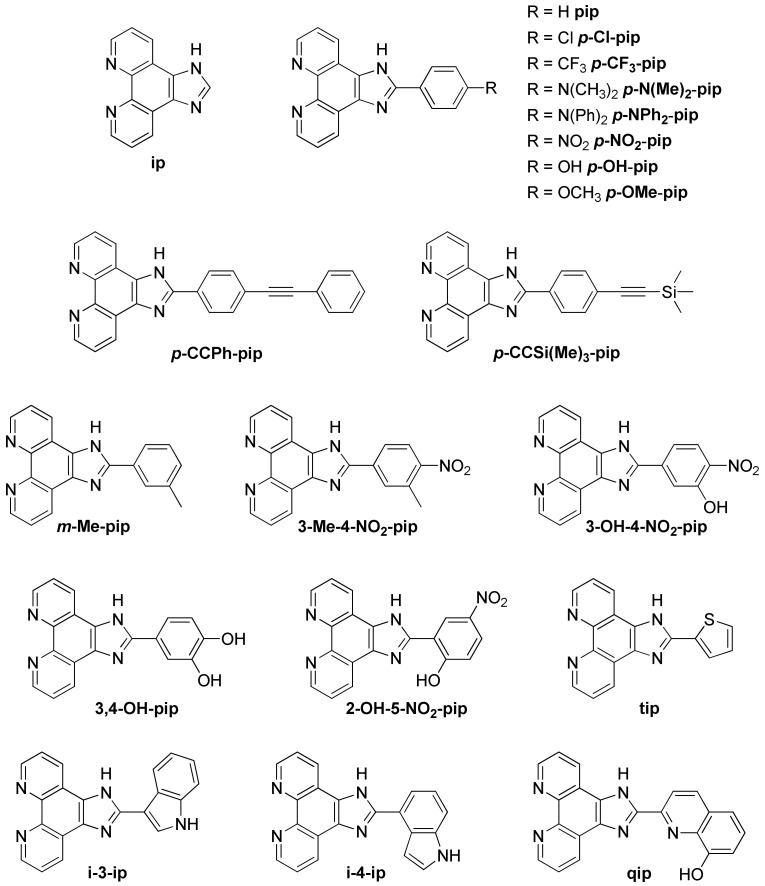
Structure of the imidazo-phenanthroline (ip) ligands and other closely related complexes used to form ruthenium(II) complexes studied in presence of G-quadruplexes.

Besides the studies involving ruthenium(II) complexes based on an extended planar ligand, other researches focused on complexes bearing a smaller moiety, the imidazo-phenanthroline. The structure of all the imidazo-phenanthroline derivatives used as ligand to form ruthenium(II) complexes studied in the presence of G-quadruplexes are presented in Figure 20.

Contrary to complexes containing a dppz-based ligand, complexes bearing an imidazo-phenanthroline ligand are luminescent in water. They consequently do not show a light-switch ON effect in the presence of DNA. Nevertheless, their luminescence still increases to various extents in presence of different DNA structures, which allows one to assess their interaction with G-quadruplexes. These complexes do show a stronger affinity towards G-quadruplexes over double-stranded DNA [226,227,228,229,230,231,232,233,234,235,236]. The small variations on the structure of the ligands does influence the binding constant of the complex with G-quadruplexes which goes from 10^4^ M^−1^ for less efficient binders ([Ru(bpy)_2_(**i-3-ip**)]^2+^) [235] to almost 10^7^ M^−1^ for complexes with high affinity ([Ru(phen)_2_(***p*-OMe-pip**)]^2+^, [Ru(phen)_2_(***p*-OH-pip**)]^2+^ or [Ru(phen)_2_(***p*-NPh_2_-pip**)]^2+^) [227,228,233]. It was shown that imidazo-phen-based complexes bearing phen ancillary ligands have a stronger affinity for G-quadruplexes than complexes with bpy ligands [226,229,230,234,237,238]. This is due to the higher hydrophobicity of phen ligands which enhances the interaction of the corresponding complexes in the grooves and loops of G-quadruplexes. The Λ-enantiomer does also show a higher binding affinity due to a more favorable interaction geometry with G-quadruplexes [227,228,231,235]. Two studies led by Liu et al. also show that having more than one imidazo-phen-based ligand does not lead to higher affinities of the complex with the G-quadruplexes as seen for [Ru(**ip**)_3_]^2+^, [Ru(**ip**)_2_(**pip**)]^2+^, [Ru(**ip**)(**pip**)_2_]^2+^ and [Ru(**pip**)_3_]^2+^ whose binding constant are 1.05 × 10^6^ M^−1^, 1.03 × 10^6^ M^−1^, 2.5 × 10^5^ M^−1^ and 4.7 × 10^5^ M^−1^, respectively [239,240].

Since these complexes do not show the light-switch ON effect desired for the design of molecular probes, they have not been studied for this kind of application. Instead, the main interest of imidazo-phen-containing complexes is their use as potential drugs for the inhibition of telomerase activity and potential agents for cancer treatment which is discussed below.

Recently, a few complexes containing a nitro group have been studied ([Ru(L)_2_(**3-OH-4-NO_2_-pip**)]^2+^, [Ru(L)_2_(**2-OH-5NO_2_-pip**)]^2+^, [Ru(bpy)_2_(***p*-NO_2_-pip**)]^2+^ and [Ru(bpy)_2_(**3-Me-4-NO_2_-pip**)]^2+^ with L = bpy or phen) [237,238,241]. These complexes show the same light-switch ON property as dppz-containing complexes and are selective towards G-quadruplex structures over double- or single-stranded DNA. This light-switch ON effect can be attributed to the highly stabilizing hydrogen bonding between water and the nitro group which is prevented when the complex interacts with G-quadruplexes.

The ability of these complexes to inhibit the action of the telomerase enzyme is mainly monitored through PCR-stop (Polymerase Chain Reaction) and TRAP (Telomerase Repeated Amplification Protocol) assays. As all the ruthenium(II) complexes containing an imidazo-phenanthroline derivative as ligand do show some binding affinity towards G-quadruplexes, they all tend to inhibit the replication of this DNA strand to some extent. However, their efficiency as potential anti-cancer drug is directly linked to their affinity for G-quadruplexes. In vitro studies to assess the cytotoxicity of these complexes for different types of cancerous cells show that depending on the type of cancerous cells, the IC_50_ value of these ruthenium(II) complexes goes from around 10 μM to a few hundred μM [226,229,232,234,235,237,239], with some complexes showing an IC_50_ comparable to that of Cisplatin as, for example, [Ru(**ip**)_2_(**pip**)]^2+^ which displays an IC_50_ of 14.4 μM against lung carcinoma epithelial cells (A549), whereas Cisplatin has an IC_50_ of 13.6 μM [237]. The complex [Ru(phen)_2_(**p-CF_3_-pip**)]^2+^ even has an IC_50_ of 16.3 μM against breast cancer cells (MDA-MB-231) which is twice as low as Cisplatin under the same conditions (IC_50_ = 36.1 μM) [229].

**Figure 21:**
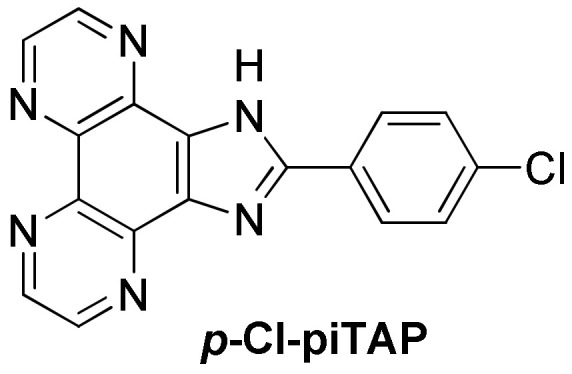
Structure of the imidazo-TAP ligand studied by Elias et al. [242].

Recently, Elias et al. reported a series of complexes based on the ***p*-Cl-pip** (2-(4-chlorophenyl)-1*H*-imidazo[4,5-*f*][1,10]phenanthroline) ligand (Figure 20) [242], aiming initially at designing new photo-oxidizing complexes by introducing TAP moieties either as ancillary ligands, by modifying the phenanthroline imidazole ligand with TAP leading to the ***p*-Cl-piTAP** (2-(4-chlorophenyl)-1*H*-imidazo[4,5-*f*]pyrazino[2,3-*h*]quinoxaline) ligand (Figure 21) or by combining both methods. The studied complexes show high affinities for G-quadruplexes, namely displaying similar affinities as that reported for BRACO-19 and are selective towards G-quadruplexes. Theoretical calculations predicted two binding modes: π-stacking over external G-tetrads and insertion of the complex into the TTA loop [242]. The high affinity towards G-quadruplexes motivated those authors to evaluate the cell penetration ability of these complexes into U2OS osteosarcoma cells. However, the luminescence in the cells was too weak to be detected by confocal microscopy except in the case of [Ru(phen)_2_(***p*-Cl-pip**)]^2+^, which revealed efficient at penetrating the cells, even inside the nucleus, after 24 h of incubation. Additionally, preliminary photo-cytotoxicity experiments were conducted on U2OS osteosarcoma cells. As expected, the [Ru(TAP)_2_(***p*-Cl-pip**)]^2+^ complex was revealed to be photo-cytotoxic by inducing 100% cell mortality under irradiation. However, [Ru(phen)_2_(***p*-Cl-pip**)]^2+^ exhibited remarkable photo-cytotoxicity as well. Therefore, it seems more likely that this effect might be due to singlet oxygen photosensitization in contrast to the typical type I photoreaction (i.e., photoinduced electron transfer (PET)) expected for TAP complexes. Additionally, this might be the first example of photo-cytotoxic ruthenium(II) complexes that does not involve the inhibition of telomerase activity, as these cancer cells do not express the telomerase enzyme. Nonetheless, further studies are necessary to give deeper insight into their photo-cytotoxicity.

**Figure 22:**
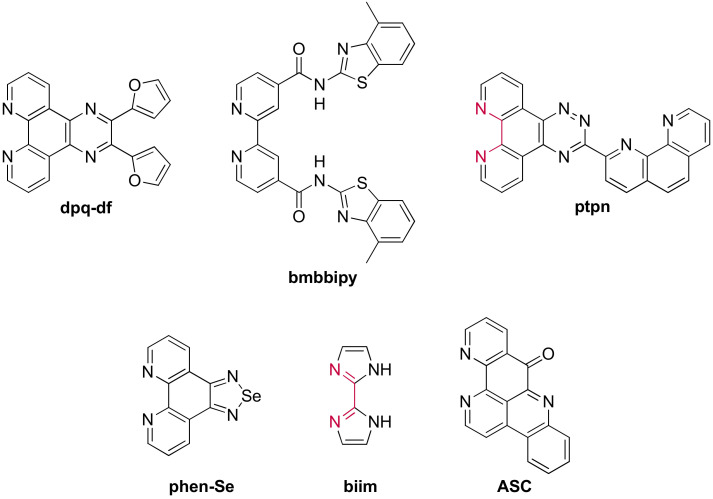
Structure of other types of ligands used to form ruthenium(II) complexes studied in presence of G-quadruplexes.

A few examples of ruthenium(II) complexes used for their interaction with G-quadruplexes do not fall in the previously discussed categories. The ligands composing these complexes are represented in Figure 22. The complexes containing the **dpq-df** (dipyrido(3,2-*a*:2′,3′-*c*)quinoxaline-difuran) or **bmbbipy** (*N,N’*-bis-(4-methyl-benzothiazol-2-yl)-2,2′-bypiridine-4,4′-dicarboxyamide) ligands do show a significant light-up effect when put in presence of G-quadruplex DNA [146,243]. The complexes based on the **ptpn** (3-(1,10-phenanthroline-2-yl)-*as*-triazino[5,6-*f*]1,10phenanthroline), **phen-Se** (1,10-phenathrolineselenazole), and **biim** (2,2′-bisimidazole) ligands do not show any significant light-up properties and have thus been studied as potential anti-cancer drugs [244,245,246]. They show similar binding affinity and cytotoxicity as the previously discussed mononuclear complexes.

**Figure 23:**
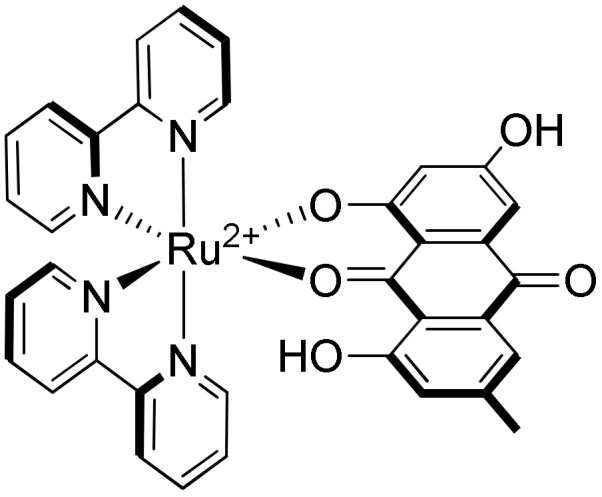
Structure of the [Ru(bpy)_2_(**emodin**)]^2+^ complex studied by Mei et al. [248].

Two biologically active molecules (**ASC** (Ascididemin) and **Emodin**) have also been used to form the [Ru(L)_2_(**ASC**)]^2+^ (L = bpy, phen or dpq) and [Ru(bpy)_2_(**emodin**)]^2+^ (Figure 23) complexes in order to combine the cytotoxic activity of the free ligands with the high binding affinity of ruthenium(II) complexes towards G-quadruplexes [247,248].

**Figure 24:**
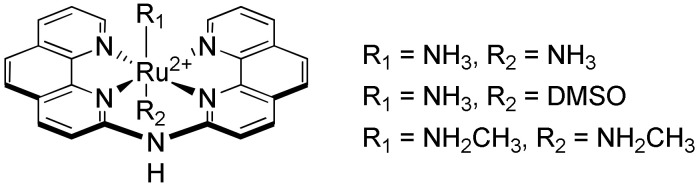
Structure of the complexes using the constrained bpa ligand studied by Shao et al. [249].

Three complexes bearing a planar tetradentate ligand composed of two phenanthroline moieties linked by an amine (Figure 24) have been synthesized by Shao et al. [249]. The new geometry obtained with these complexes allowed to obtain a higher selectivity of interaction for G-quadruplexes over double-stranded DNA, with [Ru(NH_3_)_2_(**bpa**)]^2+^ (bpa = *N,N’*-bis-(1,10-phenanthrolin-2-yl)-amine) showing a binding constant of around 4.5 × 10^5^ M^−1^ towards various G-quadruplexes whereas no binding constant could be determined due to the lack of affinity for double-stranded DNA.

The complexes discussed in this section show that the diversity of ligands used to form ruthenium(II) complexes studied for their interaction with G-quadruplexes should not be limited to dppz derivatives.

#### 3.1.4. Ruthenium(II) Complexes Bearing Schiff Base-Type Ligands

**Figure 25:**
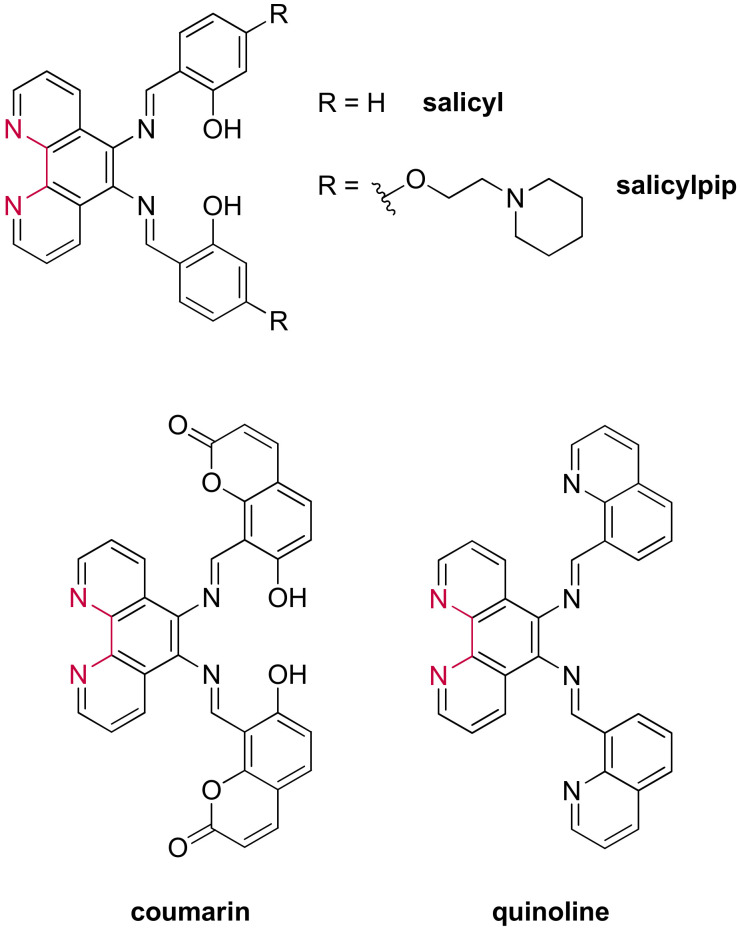
Structure of the Schiff base ligands used to form the ruthenium(II) complexes studied in the presence of G-quadruplexes.

Recent advances have revealed the importance of designing more flexible ligands to identify G4 scaffolds over duplex DNA due to favored groove interactions [250,251]. Research in designing new ruthenium(II) complexes carrying a more flexible ligand arose naturally, with the recent example of complexes carrying a Schiff base-type ligand. This type of ligand (Figure 25) was selected because it presents multiple single bonds allowing free rotational movements [252]. Additionally, the salphen moiety found in the **salicyl(pip)-** and **coumarin**-based complexes allows the possible chelation of a second metal center, which will be discussed later in this review.

These studies showed that the luminescence of the complexes significantly increases in the presence of genetic material. However, only [Ru(phen)_2_(**salicyl**)]^2+^ and [Ru(phen)_2_(**coumarin**)]^2+^ revealed to be promising at differentiating between the different DNA structures. Complementary studies by circular dichroism confirmed these results by displaying a more important stabilization effect on G4s relative to duplex DNA; therefore, displaying the selectivity of these complexes towards G-quadruplexes. It appears that the addition of positively charged piperidine chains on the salphen moiety increases the affinity towards G-quadruplexes, allowing a more favorable interaction with the grooves, already reported for the previously mentioned metal salphen complexes. An enhancement of the affinity was also observed with [Ru(phen)_2_(**coumarin**)]^2+^ and [Ru(phen)_2_(**quinoline**)]^2+^ for which the salicyl moiety has been replaced by a coumarin or quinoline moiety, respectively. This enhancement was attributed to higher π-stacking interactions through the coumarin or quinoline moiety compared to the salicyl part. Nevertheless, the affinity towards G-quadruplexes of these new Schiff base complexes is of the same order of magnitude when compared to other ruthenium(II) complexes already reported in the literature.

These compounds were evaluated in their ability to penetrate U2OS osteosarcoma cells due to their promising luminescence properties. The studied complexes penetrate the cells but with different intracellular localization (outside and inside the nucleus). Incorporating piperidine fragments leads to an increased ability of the complexes to target the genetic material, which follows the trend of the high affinity of those compounds towards G-quadruplexes. These Schiff base ruthenium(II) compounds were found to be capable of labelling the nucleoli, very dense in nucleic acids. Consequently, the cytotoxicity of these Schiff base compounds was investigated, both in the dark (IC_50_ = 47 to >100 µM) and under irradiation (IC_50_ = 0.33 to 2.1 µM). Under the experimental conditions, the complexes only exhibited very low toxicity in the dark, whereas cell viability was remarkably affected under irradiation. Surprisingly, the presence of the piperidine arms increased the photo-cytotoxicity but also induced slightly higher toxicity in the dark [252].

### 3.2. Bimetallic and Trimetallic Ruthenium(II) Complexes

#### The First G-quadruplex “Light-Switch” Ruthenium(II) Complexes

**Figure 26:**
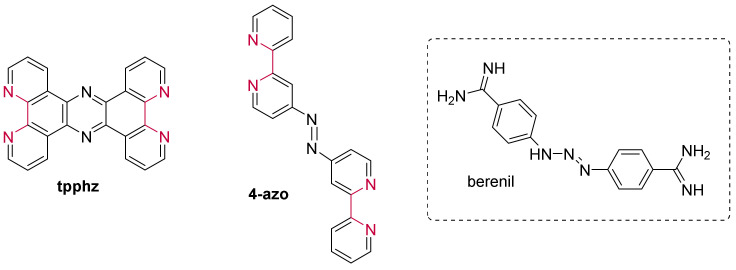
Structure of the tpphz and 4-azo ligands to form the dinuclear ruthenium(II) complexes studied by Thomas et al. in the presence of G-quadruplexes [255,256,257,258,259].

Pioneering work carried out by the Barton group that identified the “light-switch” effect of [Ru(bpy)_2_(**dppz**)]^2+^ and [Ru(phen)_2_(**dppz**)]^2+^ upon interaction with duplex DNA [253,254], has marked a milestone in the application of ruthenium(II) complexes as probes for genetic material. Following this discovery, Thomas et al. investigated the use of dinuclear Ru(II) complexes as potential G-quadruplex probes in 2006 [255]. These [{Ru(bpy)_2_}_2_(**tpphz**)]^4+^ and [{Ru(phen)_2_}_2_(**tpphz**)]^4+^ complexes incorporate the **tpphz** bridging ligand (Figure 26), with a more extended aromatic ring system than dppz, and therefore structurally reminiscent of some of the aforementioned G4-binding ligands.

These dinuclear complexes bind to G4 structures with a preference for the antiparallel human telomere sequence over duplex structures. Furthermore, an emission enhancement by ~2.5 times with a more pronounced hypsochromic shift in the presence of G-quadruplexes than observed for duplex DNA was detected [255]. This finding made these complexes the first G-quadruplex “light-switches” at that time, able to differentiate between the interaction with different DNA structures [255,256].

Further studies revealed that chirality also is a relevant factor in the interaction of the investigated complexes with the G-quadruplex structure. A combined analysis of NMR and molecular dynamics simulations demonstrated that the binding affinity of the ΛΛ-[{Ru(phen)_2_}_2_(**tpphz**)]^4+^ is 40 times higher than the ΔΔ isomer (2.95 × 10^7^ M^−1^ vs 1.16 × 10^5^ M^−1^). Only the ΛΛ isomer can interact through threading in the diagonal loop while the ΔΔ isomer binds mainly at the lateral loop [257].

Following their light-switch properties, the cytotoxicity of these complexes towards commonly studied cell cultures, such as the MCF-7 human breast cancer cells, were investigated. Neither of these dinuclear compounds display any cytotoxicity after 24 h of incubation and show an IC_50_ value of 138 µM [258]. However, [{Ru(bpy)_2_}_2_(**tpphz**)]^4+^ is only taken up by fixed cells and therefore can act as an indicator of cell mortality. On the other side, the dinuclear [{Ru(phen)_2_}_2_(**tpphz**)]^4+^ complex demonstrated a successful penetration into live cells and is located throughout the cell cytosol but found in higher concentrations within the nucleus as well as within heterochromatin. Interestingly, this compound presents two different non-colocalized emission peaks and proves to be an excellent DNA structural probe, functioning to mark duplex and G-quadruplex structures [258].

Shortly afterwards, Thomas et al. investigated the effect of an azo-based tethered ligand on the interaction of ruthenium(II) complexes with G-quadruplexes [259]. The reported compound [{Ru(bpy)_2_}_2_(**4-azo**)]^4+^ (**4-azo** = 4,4″-azobis(2,2′-bipyridine) carries a structurally similar ligand to the minor groove binder berenil and displays a colorimetric response of the buffer solution in the presence of G-quadruplexes. Further investigations are still needed to obtain a better comprehension of this phenomenon.

**Figure 27:**
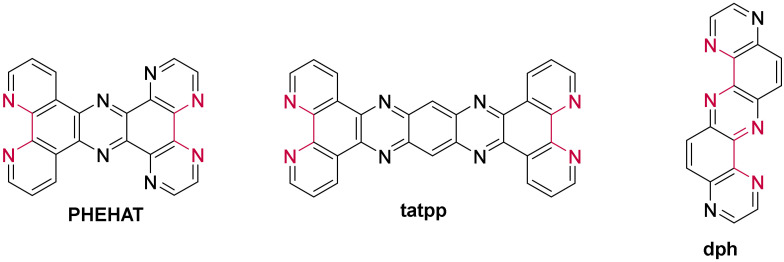
Structure of the π-extended ligands bridging ligands to form dinuclear ruthenium(II) complexes studied in the presence of G-quadruplexes.

More recently, another dinuclear complex [{Ru(phen)_2_}_2_(**PHEHAT**)]^4+^ (Figure 27), based on the π-extended **PHEHAT** bridging ligand, also revealed a “light-switch” behavior in the presence of G-quadruplexes [192]. However, in contrast to its mononuclear analogue mentioned earlier, this complex stabilizes more strongly the antiparallel conformation than the hybrid form of the human telomere sequence. Unfortunately, further studies indicated that this dinuclear complex shows no preference for neither G4 DNA nor duplex DNA despite being considered a good G4 ligand [192].

A recent publication reported a dinuclear ruthenium complex based on the **tatpp** ligand (Figure 27) [215], which displays the highest affinity (K = 4.2 × 10^7^ M^−1^) for G-quadruplexes reported up to this point for a dinuclear ruthenium compound. Similar to [{Ru(phen)_2_}_2_(**tpphz**)]^4+^ [255,256,257], [{Ru(phen)_2_}_2_(**tatpp**)]^4+^ interacts with the G4 scaffold through two binding modes, mainly by end-stacking on the G-tetrads and interaction through the grooves [215]. Through thermodynamic studies, Lewis et al. attributed these two binding modes to the preferential binding of the Λ,Λ-enantiomer over that of the other two isomers.

Due to the two equivalent chelating sites of the previously mentioned **dph** ligand, the dinuclear ruthenium(II) complex (Figure 27) was synthesized and studied in the presence of G-quadruplex structures [225]. Similar to the mononuclear analogue, the dinuclear compound displays a modest binding affinity and selectivity towards G-quadruplexes.

**Figure 28:**
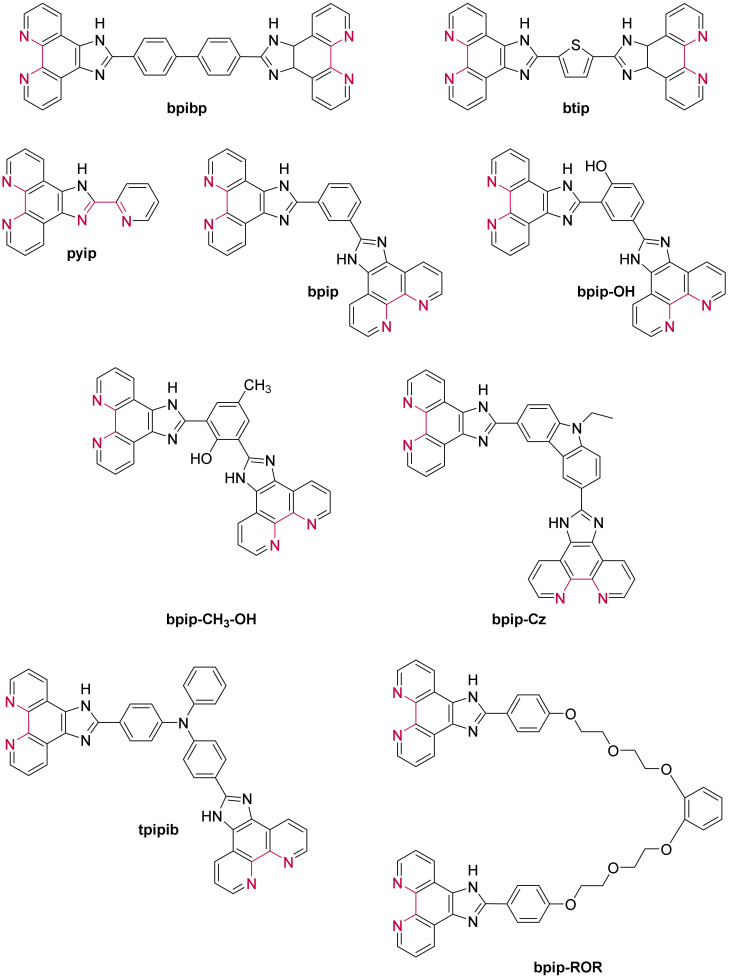
Structure of imidazo-phenanthroline ligands used to form dinuclear ruthenium(II) complexes studied in presence of G-quadruplexes.

A few dinuclear complexes based on the imidazo-phenanthroline ligand have also been investigated. The structure of the bridging ligands can be found in Figure 28. When compared to the mononuclear ruthenium(II) complexes containing one imidazo-phenanthroline ligand, these dinuclear complexes tend to have a higher binding constant. For example, [{Ru(bpy)_2_}_2_(**btip**)]^4+^ and [{Ru(bpy)_2_}_2_(**bpip-OH**)]^4+^ have a binding constant of 1.16 × 10^7^ M^−1^ and 9.07 × 10^6^ M^−1^ towards G-quadruplexes [260,261], whereas their mononuclear counter-parts [Ru(bpy)_2_(**tip**)]^2+^ and [Ru(phen)_2_(***p*-OH-pip**)]^2+^ (Figure 20) have a binding constant of 6.33 × 10^5^ M^−1^ and of around 8.5 × 10^6^ M^−1^, respectively [226,228]. While for the phenol containing imidazo-phen, the increase in the binding constant is not dramatic, for the thiophene-containing complexes the binding constant of the dinuclear complex is twice as high as for the mononuclear complex.

The only complex showing significant light-up properties is [{Ru(bpy)_2_}_2_(**bpip-ROR**)]^4+^ [262]. This light-up effect is explained by the ability of the noncyclic crown ether linking the two ruthenium(II) chromophores to interact with the K^+^ cations present in the aqueous buffered solution used, leading to a luminescence quenching. However, when the complex is in presence of G-quadruplexes, its luminescence is restored due to their interaction with the G4 structure, preventing the quenching by the K^+^ cations.

**Figure 29:**
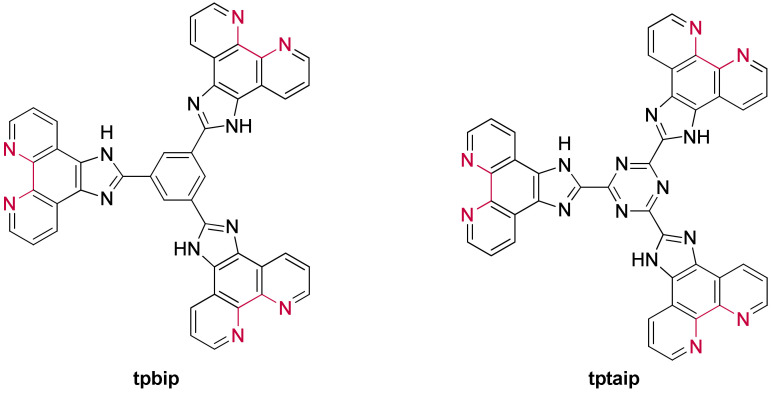
Structure of two bridging imidazo-phenanthroline ligands used to form trinuclear ruthenium(II) complexes studied for their interaction with G-quadruplexes.

No in-depth study has been carried out on the interaction of other dinuclear ruthenium(II) complexes bearing a bridging imidazo-phen ligand (**bpibp**, **pyip**, **bpip**, **bpip-CH_3_-OH**, **bpip-Cz,** and **tpipib**) with G-quadruplexes, but they have been investigated as potential anti-cancer drugs due to their ability to stabilize G-quadruplex structures [263,264,265]. Two examples of trinuclear ruthenium(II) complexes ([{Ru(bpy)_2_}_3_(**tpbip**)]6^+^ and [{Ru(bpy)_2_}_3_(**tptaip**)]6^+^) (Figure 29) have also been studied for the same purpose [266].

**Figure 30:**
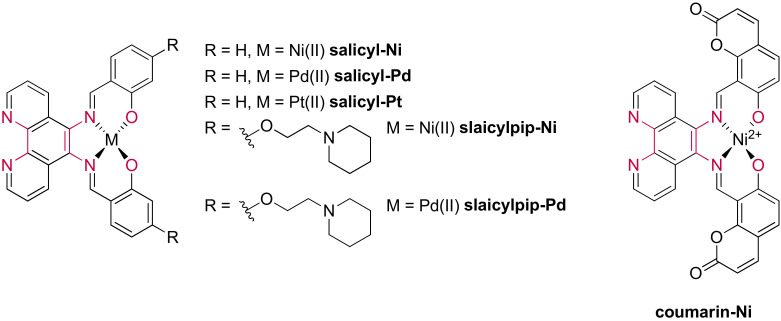
Structure of the Schiff base bridging ligands used to form the bimetallic compounds studied in the presence of G-quadruplexes.

In addition to dinuclear ruthenium(II) complexes, some bimetallic compounds were also studied in the presence of G-quadruplexes, such as those carrying a Schiff base-type ligand, previously mentioned (Figure 30) [252]. A second metal center was chelated to the salphen moiety to prevent any free rotation; therefore, leading to the stiffening of the ligand. However, the bimetallic Ru(II)-Ni(II) and Ru(II)-Pd(II) compounds are barely luminescent under the experimental conditions, making them less apt to be exploited in biological assays. Surprisingly, the Ru(II)-Pt(II) complexes exhibit much stronger luminescence alone in the solution. Furthermore, their emission is enhanced and accompanied by a hypsochromic shift once they interacted with the G4 scaffold.

Globally, the stabilization of G-quadruplexes by the bimetallic compounds appeared to be less effective when compared to their monometallic analogues. However, a small increase in stabilization can be observed when piperidine chains are added to the salphen moiety. Adding these piperidine-derived compounds also seems to follow the trend of increasing the affinity of the bimetallic complexes towards G-quadruplexes, similar to their monometallic parent compounds. Additionally, the stiffening of the Schiff base subunit by the addition of the second metal also leads to an upgraded affinity of bimetallic complexes when compared to the monometallic parent compound.

Due to the excellent luminescence properties of the Ru(II)-Pt(II) complexes, in cellulo studies were conducted with U2OS osteosarcoma cells. Similar to their mononuclear parent compounds, the bimetallic complexes can penetrate the cells to locate within the nuclei and show a weak toxicity in the dark (IC_50_ = 33 to >100 µM), but the cell viability was strongly affected under irradiation (IC_50_ = 0.73 to 2.7 µM) [252].

## 4. Photoreaction of Ruthenium(II) Complexes with G-quadruplexes

**Figure 31:**
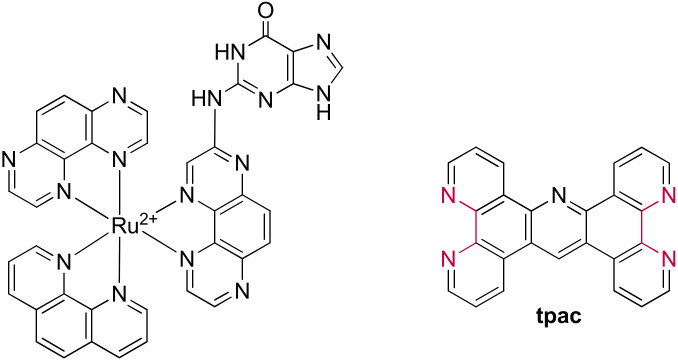
Structure of the photo-adduct of [Ru(TAP)_2_(phen)]^2+^ with a guanine base. Structure of the tpac bridging ligand used to form the dinuclear photoactive ruthenium(II) complex.

Ru(II) complexes carrying at least two π-deficient ligands, such as TAP or HAT (1,4,5,8,9,12-hexaazatriphenylene), are known to initiate a photoinduced electron transfer (PET) from a guanine base to the excited complex [267,268,269,270,271]. This PET can lead to the formation of a covalently linked adduct between the complex and the guanine, for example, as observed with [Ru(TAP)_2_(phen)]^2+^ (Figure 31) [272]. This photoreaction of ruthenium(II) complexes was also observed in the presence of G-quadruplexes, notably, the first one being with [{Ru(TAP)_2_}_2_(**tpac**)]^4+^ (tpac = tetrapyridoacridine) (Figure 31) [273].

This dinuclear complex, developed within our research group, was shown to be photoreactive and formed covalent adducts with guanine bases of both duplex and G4 DNA. The photoreactivity is mainly due to two factors: a strong interaction with the biomolecule and the independence of each ruthenium center; therefore, allowing each metallic center to keep its excited state properties, which results in the formation of several covalent bonds. After the first photon absorption and the subsequent adduct formation, the newly formed species can absorb a second photon and form a second adduct with another guanine residue, consequently inducing a photocrosslinking between both guanine units. This photocrosslinking is of particular interest since it strongly stabilizes the G4 scaffold and shows significant potential in blocking the telomerase activity. Mass spectrometry also revealed the formation of multiple photo-adducts with G-quadruplexes as well as with duplex DNA, confirming the photocrosslinking phenomenon. However, this dinuclear complex barely shows any selectivity over double-stranded DNA since the binding affinities are of the same order of magnitude (7.6 × 10^6^ M^−1^ and 2.9 × 10^6^ M^−1^ for G-quadruplex and duplex DNA, respectively) [273].

Following this discovery, Thomas et al. developed a dinuclear complex bearing π-deficient ancillary ligands and the structurally similar bridging ligand, **tpphz** (Figure 26) [274]. The TAP analogue of [{Ru(phen)_2_}_2_(**tpphz**)]^4+^, namely [{Ru(TAP)_2_}_2_(**tpphz**)]^4+^, is capable of promoting photo-oxidation of guanine sites in duplex and G4 DNA. However, the substitution of phen ancillary ligands by the π-deficient TAP ligands results in a lower affinity towards G-quadruplexes than duplexes, namely with a binding constant about an order of magnitude weaker than those set out for duplex binding. In cellulo studies of the photoactive dinuclear tpphz analogue revealed the capacity of this complex to penetrate human C8161 melanoma cells, located primarily in the nuclei. In contrast to [{Ru(phen)_2_}_2_(**tpphz**)]^4+^, the TAP derivate also produces bright emission from the cytoplasm after diffusion from the nucleus throughout the cell. Similar to the vast majority of ruthenium(II) complexes, [{Ru(TAP)_2_}_2_(**tpphz**)]^4+^ did not show any cytotoxicity in the dark, whereas it was revealed to be an efficient photosensitizer for PDT as the cell viability dropped to zero after cell irradiation [274].

## 5. Conclusions

Ruthenium(II) polypyridyl complexes have been widely studied for the last thirty years for their interaction with DNA. However, investigating these complexes to specifically interact with G-quadruplex structures has only started to gain interest in the last fifteen years. These G-quadruplex structures have yet to be fully understood but seem to play an important role as gene expression regulators as well as key targets for potential cancer treatment since they were found in telomeres. In this regard, ruthenium(II) polypyridyl complexes can play a significant role due to their remarkable behavior when interacting with G-quadruplexes. Complexes based on the dppz ligand can act as efficient light-up probes able to selectively localize the position of such G-quadruplexes. Alternatively, other complexes can act as potential anti-cancer drugs either in the dark, by hindering the replication of telomeric sequences leading to the death of tumorous cells, or under light irradiation leading to the formation of reactive oxygen species (ROS) or to the formation of photo-adducts. As shown in this review, the structure and thus the activity of ruthenium(II) complexes can be easily modified, leading to a large array of possible finetuning. However, a deep understanding of the key structural parameters influencing the interaction of these complexes with G-quadruplexes is still lacking. Therefore, the characterization of the interaction geometries of different ruthenium(II) complexes with G-quadruplexes has yet to be determined using advanced techniques such as those exploited for the [Ru(L)_2_(**dppz**)]^2+^ complexes. This should allow the design of new highly selective tools for the recognition, study, and reversible or irreversible marking of G-quadruplexes, which might lead to exciting discoveries in biomedical sciences.
